# Culture shock: microglial heterogeneity, activation, and disrupted single-cell microglial networks in vitro

**DOI:** 10.1186/s13024-022-00531-1

**Published:** 2022-03-28

**Authors:** Mika P. Cadiz, Tanner D. Jensen, Jonathon P. Sens, Kuixi Zhu, Won-Min Song, Bin Zhang, Mark Ebbert, Rui Chang, John D. Fryer

**Affiliations:** 1 Department of Neuroscience, Mayo Clinic, Scottsdale, AZ 85259 USA; 2 Neuroscience Graduate Program, Mayo Clinic Graduate School of Biomedical Sciences, Scottsdale, AZ 85259 USA; 3 Department of Neurology, University of Arizona, Tucson, AZ 85721 USA; 4 Department of Genetics & Genomic Sciences, Mount Sinai Center for Transformative Disease Modeling, Icahn School of Medicine at Mount Sinai, New York, NY 10029 USA; 5 Sanders-Brown Center on Aging, Biomedical Informatics, and Department of Neuroscience, University of Kentucky, Lexington, KY 40536 USA

**Keywords:** Microglia, In vitro culture, Single cell RNA sequencing, Causal network analysis

## Abstract

**Background:**

Microglia, the resident immune cells of the brain, play a critical role in numerous diseases, but are a minority cell type and difficult to genetically manipulate in vivo with viral vectors and other approaches. Primary cultures allow a more controlled setting to investigate these cells, but morphological and transcriptional changes upon removal from their normal brain environment raise many caveats from in vitro studies.

**Methods:**

To investigate whether cultured microglia recapitulate in vivo microglial signatures, we used single-cell RNA sequencing (scRNAseq) to compare microglia freshly isolated from the brain to primary microglial cultures. We performed cell population discovery, differential expression analysis, and gene co-expression module analysis to compare signatures between in vitro and in vivo microglia. We constructed causal predictive network models of transcriptional regulators from the scRNAseq data and identified a set of potential key drivers of the cultured phenotype. To validate this network analysis, we knocked down two of these key drivers, *C1qc* and *Prdx1,* in primary cultured microglia and quantified changes in microglial activation markers.

**Results:**

We found that, although often assumed to be a relatively homogenous population of cells in culture, in vitro microglia are a highly heterogeneous population consisting of distinct subpopulations of cells with transcriptional profiles reminiscent of macrophages and monocytes, and are marked by transcriptional programs active in neurodegeneration and other disease states. We found that microglia in vitro presented transcriptional activation of a set of “culture shock genes” not found in freshly isolated microglia, characterized by strong upregulation of disease-associated genes including *Apoe*, *Lyz2,* and *Spp1*, and downregulation of homeostatic microglial markers, including *Cx3cr1*, *P2ry12*, and *Tmem119*. Finally, we found that cultured microglia prominently alter their transcriptional machinery modulated by key drivers from the homeostatic to activated phenotype. Knockdown of one of these drivers, *C1qc*, resulted in downregulation of microglial activation genes *Lpl, Lyz2, and Ccl4.*

**Conclusions:**

Overall, our data suggest that when removed from their in vivo home environment, microglia suffer a severe case of “culture shock”, drastically modulating their transcriptional regulatory network state from homeostatic to activated through upregulation of modules of culture-specific genes. Consequently, cultured microglia behave as a disparate cell type that does not recapitulate the homeostatic signatures of microglia in vivo. Finally, our predictive network model discovered potential key drivers that may convert activated microglia back to their homeostatic state, allowing for more accurate representation of in vivo states in culture. Knockdown of key driver *C1qc* partially attenuated microglial activation in vitro, despite *C1qc* being only weakly upregulated in culture. This suggests that even genes that are not strongly differentially expressed across treatments or preparations may drive downstream transcriptional changes in culture.

**Supplementary Information:**

The online version contains supplementary material available at 10.1186/s13024-022-00531-1.

## Background

Microglia, the resident immune cells of the central nervous system (CNS), play a critical role in regulating their microenvironment and maintaining brain homeostasis [1]. Unlike other peripheral monocyte populations that originate from bone marrow, microglia originate from the yolk sac at embryonic day 9.5 in mice [2] and infiltrate the brain before the closure of the blood-brain barrier. Because of their high degree of responsiveness and diverse roles in CNS homeostasis and immunity, microglia have been implicated in a variety of diseases, including Alzheimer’s disease (AD), amyotrophic lateral sclerosis/frontotemporal dementia (ALS/FTD), Parkinson’s disease (PD), and multiple sclerosis (MS), as well as brain tumors, stroke, traumatic brain injury, etc. [1].

In the healthy brain, microglia are predominantly homeostatic and possess a ramified morphology, with long processes that continuously sense the microenvironment. This phenotype is often described as ‘resting’, even though these homeostatic microglia are highly mobile and dynamic. In disease states, they take on ‘activated’ features characterized by rapid proliferation, migration, antigen presentation to infiltrating immune cells, and secretion of proinflammatory cytokines, chemokines, and reactive oxygen species [1]. Microglial activation, rather than being a singular homogenous cell state, encompasses a spectrum of heterogeneous phenotypes that vary according to disease or insult types [3, 4]. For instance, disease-associated microglia (DAM), that have been identified in AD and ALS mouse models and aging mice, are characterized by amoeboid morphology, phagocytosis of amyloid plaques, upregulation of disease genes including *Apoe*, *Trem2*, *Cst7*, and *Lpl*, and downregulation of homeostatic genes including *Tmem119*, *Hexb*, *Cx3cr1*, and *P2ry12* [5, 6]. In a meta-analysis, Friedman et al. [4] identified a set of distinct gene signatures corresponding to various insults and diseases including neurodegenerative disease, lipopolysaccharide (LPS) administration, and glioma.

Transduction of microglia with viruses in vivo is often unsuccessful or very poorly efficient [7], requiring time-consuming conditional knockout approaches with a variety of microglial-specific Cre drivers. As a result, a common alternative to in vivo studies is to use in vitro techniques with primary microglial cells, cell lines, microglia derived from induced pluripotent stem cells (iPSCs) or embryonic stem cells (ESCs), organotypic brain slice cultures, and organoids. However, N9 and BV2, two common microglial cell lines, as well as primary cultured microglia derived from P1 and P2 mice, lack the molecular signatures of adult microglia in vivo, including key homeostatic genes *Tmem119*, *P2ry12*, *Cx3cr1*, and *Hexb* [8]. Furthermore, while microglia in organotypic brain slice cultures resemble in vivo microglia more than primary cultured microglia, about 50% of these cells are non-homeostatic (either proliferative, inflammatory, or reactive), and overexpress markers associated with microglia in neurodegenerative disease [9]. In general, microglia across in vitro systems suffer from disseminated molecular changes attributed to loss of signaling with other cells in the brain microenvironment that is required to maintain them in a homeostatic state.

To characterize the subpopulations and heterogeneity in cultured microglial cells, we used single-cell RNA sequencing (scRNAseq) to profile the transcriptomes of microglia freshly isolated from the brain of wild-type C57BL/6 J mice and compared them to scRNAseq data generated from primary microglia cultures which are derived from astrocyte-microglial co-culture and microglial monocultures that have been shaken off the astrocyte co-culture, as this is still one of the most commonly used methods for primary microglial culture. We found that microglia from both co-culture and monoculture systems possess significant activation signatures compared to freshly isolated microglia, with highly heterogeneous subpopulations of cells characterized by genes involved in interferon response, proliferation, phagocytosis, autophagy, and other pathways and biological processes associated with various diseases and activation conditions. To further identify the key drivers of microglial heterogeneity and phenotypes in culture, we applied predictive network modeling, a cutting-edge causal network modeling approach, to reconstruct genetic regulatory networks of microglia in mono- and co-cultures, and identified a set of robust key drivers that could potentially perturb microglial activation in vitro to more closely mimic the in vivo homeostatic state. Our results add to growing evidence that in vitro microglia do not recapitulate the phenotype of their in vivo counterparts, and our predicted key drivers provide potential targets for restoring the homeostatic phenotype in culture. It is crucial that data from in vitro studies of microglia be interpreted in light of not only the significant activation of these cells in vitro, but also the extensive heterogeneity that suggests a non-uniform response of microglial subpopulations to perturbations or insults.

## Methods

### Mice

C57BL/6 J female mice were used. All studies were done in accordance with National Institutes of Health Guide for the Care and Use of Laboratory Animals under an approved protocol from the Mayo Clinic Institutional Animal Care and Use Committee. Two mice were harvested at ~ 4 months of age for the freshly isolated in vivo microglial preparation, and two mice each were harvested at postnatal day 1 (P1) for the astrocyte-plated and microglia-isolated cultures.

### Primary culture microglia preparation

Brains were extracted from mice, meninges were removed, and cortical tissue was dissociated by trituration with a P1000 pipette. The resulting cells were used to seed a glial co-culture. Cells were cultured in DMEM with 10% FBS and 1% penicillin-streptomycin. The following day, media was removed, and fresh media was added. Cells were cultured for 7-9 days, and at the point of confluency, microglia for the micro-isolated monoculture were removed from the culture by shaking on a plate shaker at 400 rpm until cells entered the solution. These microglia were then re-plated as a monoculture. At day 11, microglia in the monoculture were scraped off with a cell scraper, collected, and immediately used for scRNAseq. At the same time point, microglia in the mixed glial culture (“astro-plated preparation”) were removed from culture by shaking at 400 rpm and prepared for sequencing.

### Freshly isolated microglia preparation

Mice were anesthetized with pentobarbital and transcardially perfused with PBS. Brains were removed from mice, meninges were removed, and cortex was dissected and isolated and immediately put in ice cold Accutase. Cortical tissue was dissociated for 30 min at 4 °C while gently rocking. The resulting solution was centrifuged at 300 x g for 10 min, and supernatant was removed. The pellet was resuspended in 10 ml of HBSS, and cells were triturated sequentially with a 10 mL serological pipette, 5 ml serological pipette, and P1000 pipette. Cells were again centrifuged at 300 x g for 10 min, supernatant was removed, and cells were resuspended in Miltenyi myelin depletion buffer. Myelin was depleted with magnetic beads according to manufacturer’s instructions.

### Library preparation, barcoding, and sequencing

Cells were counted and processed for library preparation according to manufacturer’s instructions (10X Genomics). Cells were loaded onto Chromium Single Cell A Chip with reverse transcriptase master mix, gel beads, and partitioning oil. Gel beads in emulsion (GEMs) were generated. cDNA was purified with DynaBeads and library quantification was performed with KAPA universal library quantification kit and high sensitivity DNA kit. Samples were sequenced on a HiSeq 4000 flow cell. Reads were aligned to the mm10 mouse reference genome then aggregated and normalized using CellRanger (10X Genomics).

### Astro-plated in vitro cleaning and analysis

Raw count matrices from the pre-processed and aligned data were read into R and analyzed with the package Seurat version 3.1 (Satija et al., 2015). An initial round of cleaning and clustering was performed. Cells with more than 50,000 total molecules (nCount_RNA) and where more than 10% of detected counts originated from mitochondrial transcripts (percent.mito) were filtered out. SCTransform was used to normalize and scale data and to regress out percent.mito. PCA dimensionality reduction was performed with the first 50 principal components, and a shared nearest neighbor (SNN) graph was constructed using 30 dimensions. Clusters were identified using the Louvian clustering algorithm at a resolution of 0.6, and a graph of all cell populations was generated through uniform manifold approximation and projection (UMAP) using 30 principal components.

A second round of cleaning was performed to remove cells with percent.mito > 5, nCount_RNA < 3000, and nFeature_RNA > 6000. Additionally, clusters 6, 7, 8, 9, and 12 were removed from the dataset due to ribosomal/mitochondrial contamination, and low read counts (suggestive of empty GEM droplets). A second round of clustering was performed as previously described, and clusters composed of myeloid-like cells were identified based on expression levels of *Hexb*, *Trem2*, *C1qa*, and *Ctss*. Non-myeloid cells (astrocytes, mural cells, neural progenitors, oligodendrocyte precursor cells, oligodendrocytes) were removed from the dataset for the purposes of this manuscript and cells were re-clustered at a resolution of 0.2.

### Gene set analysis

Non-overlapping gene lists corresponding to seven different microglial states (proliferation, macrophage, interferon-related, LPS-related, monocyte, resting microglia and neurodegeneration) were identified from Friedman et al., Kang et al., and Van Hove et al. [4, 6, 10]. The PercentageFeatureSet function was used to determine the percent of transcripts from each module. Microglial subpopulations were then categorized as either proliferation, macrophage-like, monocyte-like, or microglia-like based on the percent expression of each module in each cluster.

### Freshly isolated microglia cleaning and analysis

Cells with nCount_RNA > 10,000 were removed from the dataset. An initial round of clustering was performed as previously described. Clusters containing non-myeloid cells (neurons, astrocytes, endothelial cells, oligodendrocytes, macrophages, red blood cells, neural stem cells, oligodendrocyte precursor cells, choroid cells) were identified with canonical markers and removed from further analysis for the purposes of this study. A second round of clustering and cleaning was performed at a resolution of 0.6 and cells with percent.mito > 10 were removed, and cells were re-clustered a final time, resulting in a six subpopulations of microglia. These subpopulations were classified as either homeostatic, activated, or interferon based on the marker expression of each cluster.

### Micro isolated in vitro cleaning

Cells with nCount_RNA > 10,000 and percent.mito > 20 were removed from the dataset. Clustering was performed as previously described. Cell types were identified with canonical markers, and all non-myeloid cells (identified by lack of expression of *Trem2*, *Tyrobp*, *Ctss*, and *Hexb*) were removed. More stringent filtering was performed to exclude all cells with percent.mito > 5, and cells were re-clustered at a resolution of 0.6.

### Seurat CCA integration

Samples were aggregated in the following order: astro-plated, micro-isolated, freshly isolated. Integration anchors were determined with anchor.features set at 3000, and at a dimensionality of 30. Following integration, the six samples were clustered at a dimensionality of 30 and resolution of 0.5.

### Combined differential markers testing

The FindMarkers function was used to select genes that were differentially expressed between astro-plated and freshly isolated, micro-isolated and freshly isolated, and micro-isolated and astro-plated samples. Only genes with an adjusted *p*-value of less than 0.05 and minimum average log fold change of 0.5 for the astro vs. freshly isolated and micro vs. freshly isolated, and 0.3 for the micro-isolated vs. astro-plated samples were included. The data from these gene lists was used to build volcano plots and heat maps for pseudo-bulk RNAseq analysis, and to identify shared differentially regulated genes across cultured preparations.

### Metascape gene enrichment analysis

Metascape gene annotation and analysis [11] was used to identify pathways that are differentially regulated in astro-plated vs. freshly isolated and micro-isolated vs. astro-plated samples. Gene lists were obtained as described in ‘combined differential markers testing’.

### Multiscale embedded gene co-expression network analysis (MEGENA) network analysis

Cells were downsampled to 400 cells per cluster for both freshly isolated and astro-plated preparations to prevent over-representation of homeostatic microglia from the freshly isolated preparation in the gene network. This subsample was then scaled and normalized before analysis in MEGENA [12]. Genes that were expressed in less than 10 cells in this subset were excluded from the network. Weighted module expression was calculated from the sum of the expression of each gene in the module, weighted by its strength in the network, and signed by whether that gene has positive or negative correlation with the largest hub of that module. Genes in the network are displayed as nodes, while spokes connecting nodes represent correlation between genes, suggesting two genes are co-regulated. The top 20 genes ranked by strength, the sum of all significant correlation edges from a gene node, are shown in the network. Genes of greater strength are displayed larger, while ‘hub genes’, which are marked by significantly more connections than other genes in the same neighborhood, are enclosed in diamonds.

### Single-cell predictive network construction

We applied our recently developed top-down and bottom-up predictive network pipeline to build causal predictive network models [13–16], which leverage the bottom-up belief propagation engine [17, 18] as a sub-routine to infer causality among equivalent structures. To build a predictive network from scRNAseq data, we evaluated the variance of expression level of every gene in the residual after scRNAseq data QC, normalization and covariate adjustment and then prioritized genes with expression variance in descending order to extract a list of top-varying genes from the scRNAseq residual [16]. We applied the predictive network pipeline [19–21] to construct the causal network model of top-varying genes in astro-plated and micro-isolated samples.

### Key driver discovery

We used three categories of effector gene sets for each network: 1) significant DE genes between in vitro microglia and freshly isolated microglia, 2) gene co-expression modules (9 modules for astro-plated and 6 modules for micro-isolated derived from MEGENA), 3) overlap of the significant DE genes with every gene module per network. In total we derived 291 and 169 sets of downstream effector genes for astro-plated and micro-isolated network, respectively.

### Key driver validation by reverse transcription and quantitative PCR (RT-qPCR)

A mixed glial co-culture was prepared as described in the Primary Culture Microglia methods section. Cells were plated directly in two 12-well plates per mouse brain. Media was changed every 2 days. At 9 days in culture, each well was transfected with 20 pmol total siRNA targeting either *C1qc* (Sigma siRNA IDs SASI_Mm01_00130820, SASI_Mm01_00130822), *Prdx1* (Sigma siRNA IDs SASI_Mm02_00319768, SASI_Mm02_00319769), or a non-template negative control (Qiagen 1,022,076), using Lipofectamine 3000 Transfection Reagent (Fisher L3000015). RNA was harvested 3 days post-transfection using a Qiagen RNeasy Kit, according to manufacturer’s instructions.

cDNA was generated from RNA using iScript™ Reverse Transcription Supermix (Bio-rad) according to manufacturer’s instructions. RT-qPCR reactions were set up with iTaq Universal Sybr Green Supermix (Bio-rad), 400 nM primer mix, and 100 ng cDNA template per 20 ul reaction. Amplification was performed on a Roche LightCycler480 System. Statistical analysis was performed with GraphPad Prism.

## Results

### Heterogeneity of primary cultured microglia

**Fig. 1:**
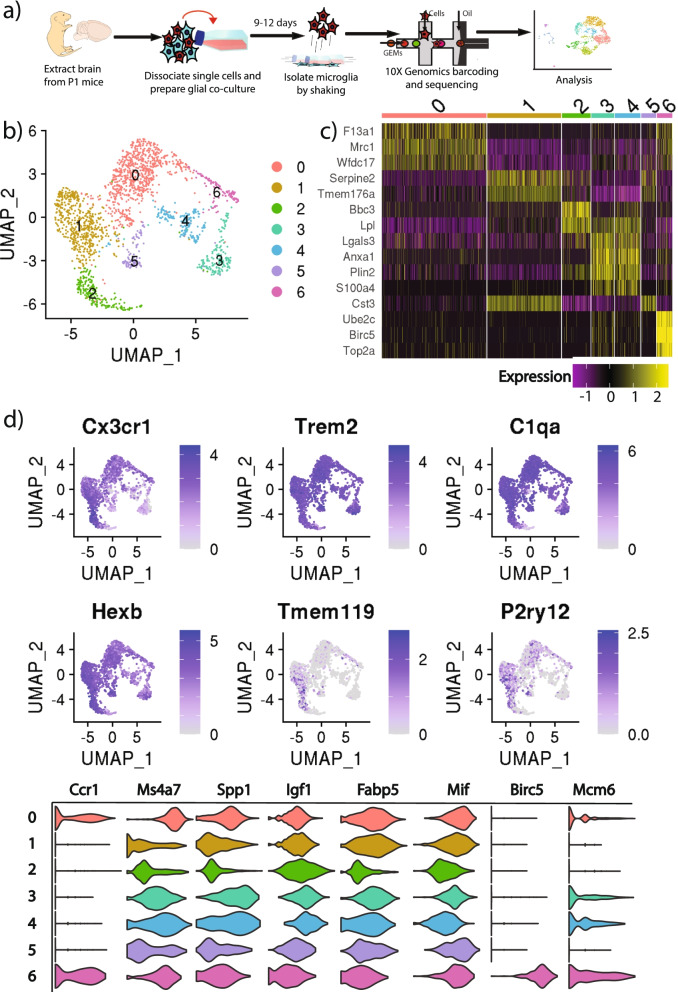
Clustering and differential expression analysis reveal substantial heterogeneity of primary culture microglia. **a** Flowchart of the experimental procedure used to generate data. Brain cortices was extracted from P1 mice. Single cells were dissociated from brain tissue and a glial co-culture was prepared. At the point of confluency, microglia were shaken off, which were used for single-cell barcoding using 10X Genomics 3′ Chromium v3. Libraries were prepared and sequenced, then the data were computationally analyzed with the Seurat package. **b** UMAP embedding and clustering of 4855 Myeloid cells reveals 7 distinct clusters of primary culture microglia. Data were downsampled to 2000 cells for visualization. **c** Heatmap showing the expression in each cell of the top differentially expressed genes that mark each cluster. Heatmap contains only the 2000 downsampled cells. **d** Feature plot of canonical microglia and myeloid markers. Cells are colored based on normalized expression value for each gene. Robust expression of shared myeloid markers is seen. However, expression of microglia-specific markers like *Tmem119* is sparse. Violin plots show that the cultured cells robustly express markers of microglial heterogeneity in the developing brain; however, these markers are broadly expressed across many clusters as opposed to in a cluster-specific manner as seen in freshly isolated developing microglia

To investigate the transcriptome of unstimulated primary cultured microglia, we performed single-cell RNA sequencing (scRNAseq) of cells freshly shaken off of a confluent glial co-culture (Fig. 1A). An initial round of clustering and differential expression analysis of the 4994 captured high-quality cells using the analysis package Seurat (Satija et al., 2015), found that 97.2% of cells were myeloid based on the expression of canonical myeloid markers (*Trem2*, *Hexb,* and *C1qa*). The remaining cells were in clusters that expressed markers of astrocytes (*Aqp4* and *Gja1*), mural cells (*Tagln* and *Acta2*), neural progenitors (*Meg3*, *Syt1,* and *Stmn3*), oligodendrocyte precursor cells (*Cspg5* and *Olig1*), and mature oligodendrocytes (*Plp1* and *Mog*). These data suggest that there is some contamination of non-microglia cell types in “microglia” that are freshly shaken off of glial co-cultures.

After removing these non-myeloid populations and filtering out low-quality and artifactual barcodes, we re-clustered the remaining 4855 cells and found cells clustered into seven heterogeneous and distinct subpopulations (Fig. 1B). Using the FindMarkers function in Seurat, we identified differentially expressed (DE) gene signatures for each cluster and discovered that this heterogeneity is driven by genes not typically expressed by resting microglia. For instance, cluster 0 differentially expressed *F13a1* a marker unique to brain-boundary or infiltrating macrophages, cluster 2 highly expressed *Lpl*, typically only expressed by activated microglia, clusters 3 and 4 expressed unique markers of peripheral monocytes like *Anxa1* and *S100a4*, and cluster 6 expressed cell cycle markers *Birc5* and *Top2a*, only expressed by actively proliferating microglia (Fig. 1C). The complete differential expression signature for every cluster of primary cultured microglial cells is listed in the Supplementary Table S1. Next, we examined whether these cells were indeed microglia since we detected robust expression of canonical myeloid markers *Trem2, C1qa,* and *Hexb.* We found that the expression of the canonical homeostatic microglial markers *Cx3cr1, P2ry12,* and particularly *Tmem119*, was sparse (Fig. 1D), suggesting that cultured microglia, while still myeloid, lose the molecular profile specific to in vivo homeostatic microglia.

Since cultured microglia were extracted from the still-developing brains of early postnatal pups, we sought to determine whether the heterogeneity of our cultured microglia arose from the heterogeneity of microglia in the developing brain. We plotted markers associated with microglia in the developing mouse brain from Hammond et al. 2018 [22] and found that most markers of developmental diversity are robustly expressed across all clusters rather than in a cluster-specific fashion, except macrophage cluster (cluster 0) and proliferative cluster (cluster 6) (Fig. 1D). These data suggest that our primary cultured microglia show limited signal of developmental diversity, which is insufficient to explain the extensive heterogeneity in these cultured cells.

### Characterization of in vitro astrocyte-plated microglial clusters by known microglial states

**Fig. 2:**
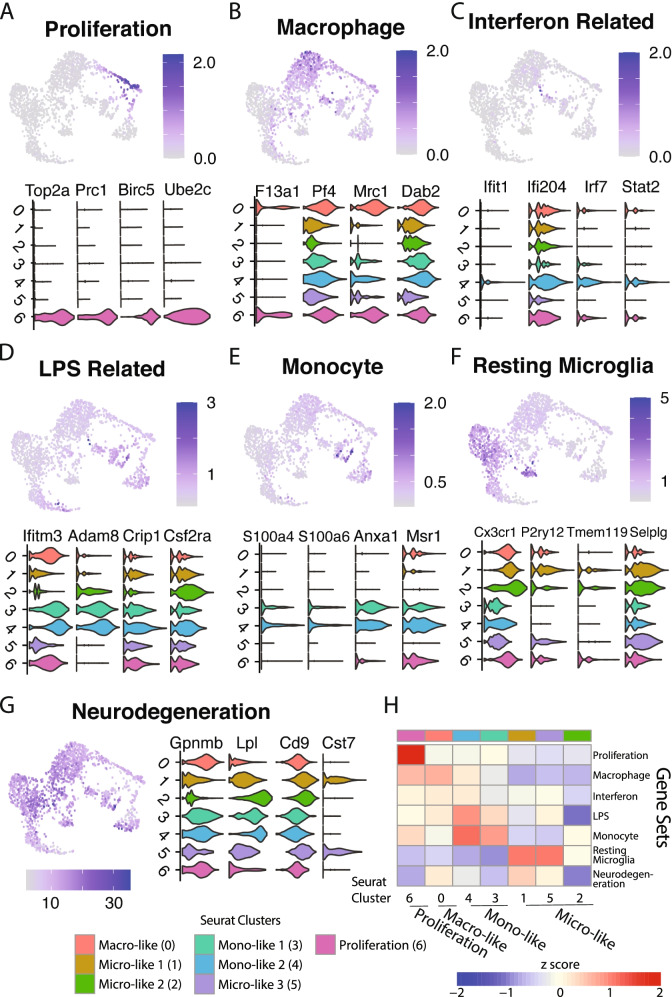
Cultured microglia recapitulate the transcriptomic profiles of CNS myeloid cells in a range of conditions. A recent meta-analysis by Friedman et al. of multiple microglia gene expression datasets revealed co-regulated gene modules expressed by myeloid cells in a variety of conditions. The extent to which these co-regulated modules could describe the heterogeneity of in vitro microglia was tested by treating each module as a gene set and calculating the percentage of UMIs in each cell that originated from each gene set. **a** Proliferation genes are cell cycle genes that are expressed by actively dividing microglia and are strongly enriched in cluster 6. **b** The macrophage gene set contains unique markers of brain macrophages when compared to parenchymal microglia and is expressed at low levels in almost every cluster, though most strongly concentrated in clusters 0 and 6. **c** The Interferon related gene set contains genes upregulated as part of the interferon signaling pathway and is expressed at low levels in parts of cluster 4. **d** The LPS related gene set contains genes that respond to LPS stimulation and is expressed at low levels in many clusters, most strongly in cluster 4. **e** Markers that uniquely characterize monocytes compared to microglia and other macrophages make up the Monocyte gene set, and are expressed by a subset of cells in clusters 3 and 4. **f** The resting microglia gene set contains homeostatic microglia markers that uniquely define microglia and is expressed in clusters 1,2, and 5. **g** Activation markers of microglia seen in a variety of neurodegenerative disease are contained in the Neurodegeneration module. There is robust neurodegeneration expression across all clusters, highlighting the heightened activation profile of cultured microglia, and the signal is strongest in cluster 1. **h** Heatmap showing the average scaled gene set percentage of each cluster allow the classification of Seurat clusters into specific module-related classes

 To further characterize the diversity of subpopulations in our cultured microglia data, we performed a gene set enrichment analysis of our microglial clusters against the known brain myeloid cell states of murine microglia that are observed under a variety of conditions, including neurodegeneration [5, 23–26], LPS injection [6], viral activation with LCMV [27], glioma [28], and different myeloid cell types [10, 29, 30]. We identified 7 non-overlapping sets of marker genes that define different brain myeloid cell states: proliferation, macrophage, interferon-related, LPS-related, monocyte, resting microglia, and neurodegeneration genes (Table 1). The percentage of transcripts in each cell from genes in the 7 different gene sets allowed us to visualize the extent of expression of these modules across cultured subpopulations. The proliferation gene set, that included cell cycle markers *Top2a* and *Birc5*, was highly and uniquely enriched in cluster 6 (Fig. 2A). The macrophage gene set, that included the markers *F13a1* and *Mrc1*, was most enriched in cluster 0 but also present at lower levels in all of the other clusters (Fig. 2B). Markers upregulated in response to virus (*Ifit1, Irf7*) and LPS-injection (*Ifitm3*, *Crip1*) seen in the interferon related and LPS related gene sets, were both lowly expressed across most subpopulations, but were most enriched in clusters 3 and 4 (Fig. 2C-D). The monocyte gene set containing *S100a4* and *Anxa1* was also uniquely expressed in a subset of cells of clusters 3 and 4 (Fig. 2E). The resting microglia gene set was mostly expressed on the left side of the UMAP in clusters 1, 2, and 5, and largely absent in the other clusters (Fig. 2F). Finally, the neurodegeneration gene set that contained the canonical markers of activated microglia such as *Lpl* and *Cst7* was robustly expressed at high levels across all clusters, though most strongly in cluster 1 (Fig. 2G). Hierarchical clustering of the average scaled expression of the gene sets across these 7 subpopulations is displayed as a heatmap that classifies these clusters into more descriptive families based on these gene sets of known brain myeloid state (Fig. 2H).

**Table 1:** Modules of co-regulated genes capturing diversity of microglia are robustly detected in in vitro cells

Gene Set	Description	Citation	Size	Number of Genes detected	Percent Detected
PROLIFERATION	Cell cycle genes marking actively proliferating microglia	Friedman et al., 2018	81	77	95.1%
MACROPHAGE	Unique markers of infiltrating and brain-boundary macrophages compared to microglia	Friedman et al., 2018 Hove et al., 2019	28	26	92.9%
LPS-RELATED	Genes upregulated in brain myeloid cells as a response to LPS injections and system inflammation	Friedman et al., 2018 Kang et al., 2018	86	75	87.2%
INTERFERON-RELATED	Genes upregulated in brain myeloid cells as a response to LCMV and Poly(I:C) injections	Friedman et al., 2018 Kang et al., 2018	52	52	91.2%
MONOCYTE	Unique markers of peripheral monocytes compared to other brain myeloid cell types	Friedman et al., 2018 Hove et al., 2019	82	64	78.0%
RESTING MICROGLIA	Unique markers of parenchymal microglia compared to other brain myeloid cell types	Friedman et al., 2018 Hove et al., 2019	50	49	98.0%
NEURODEGENERATION	Genes upregulated in the activation response of microglia seen in a variety of neurodegeneration models	Friedman et al., 2018	126	121	96.0%

GENE SET defines the name of each gene module. DESCRIPTION gives a short description to describe the genes contained. CITATION lists the sources which were used to create the gene set. SIZE gives the number of genes in the gene set. NUMBER OF GENES DETECTED lists how many genes in the gene set were detected in the cleaned myeloid in vitro cells (expressed in at least 10 cells). PERCENT DETECTED gives the number of detected genes as a percent of total gene set size. We see that with the exception of the Monocyte and LPS-related gene set—which had 78.0 and 87.2% respectively—more than 90% of genes from most gene sets were expressed

### Freshly isolated microglia form a largely homeostatic population, with a small percentage of activated cells

**Fig. 3:**
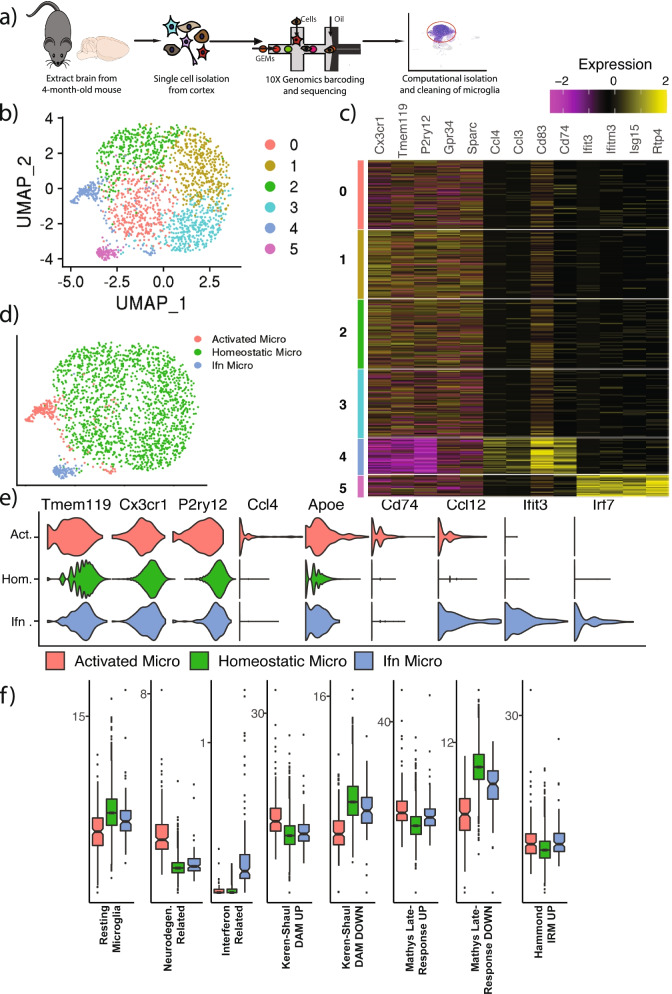
The majority of freshly isolated microglia are homeostatic with minor activated and interferon subpopulations. **a** Schematic diagram of the cell isolation and scRNAseq procedure used to generate this data. **b** UMAP and clustering of 8794 microglial cells. Clusters 0,1,2 and 3 appear to form a homogenous blob, while the much smaller clusters 4 and 5 are distinctly separate. 2000 downsampled cells are shown for visualization purposes. **c** Heatmap of the top differentially expressed genes in each cluster. Clusters 0-3 show higher expression of resting microglia markers like *Tmem119*, cluster 4 shows expression of canonical activation markers like *Ccl4*, and cluster 5 shows expression of interferon-related genes like *Ifit3*. **d** Informed by the differential expression analysis, cells are labeled as either homeostatic, activated, or interferon microglia and replotted on the UMAP embedding. **e** Violin plots showing 3 canonical markers of homeostatic, activated, and interferon-response microglia validate our classification of cells into labeled clusters. Act. = Activated, Hom. = Homeostatic, Ifn. = Interferon. **f** To validate the activated microglia cluster represents the same disease-associated microglia signature (DAM) found in mouse models of neurodegeneration, a gene set analysis was run using previously published gene sets known to be changed during activation. Percentage of UMIs belonging to genes in each gene set were calculated for each cell. Ridged box plots show the distribution of percentages between each group. A two-tailed t-test was used to compare gene set percentages between the activated cluster and the homeostatic cluster, and between the interferon cluster and the homeostatic cluster. Bonferroni correction was applied to resulting *p*-values to control for multiple testing. *P*-values and statistics from this test are presented in Table 2

To compare the transcriptome of the astrocyte-plated cultured microglia to microglia in vivo, we profiled cells freshly isolated from non-transgenic, 3.5-4-month-old mice. Whole cortices from two mice were mechanically and enzymatically dissociated then stripped of myelin, and the resulting cell suspension was prepared and sequenced using the 10X Genomics Chromium V3 protocol (Fig. 3A). After an initial round of clustering and cleaning, 8794 cells were determined to be microglia based on the expression of microglia markers *Cx3cr1*, *P2ry12,* and *Trem2*. These cells were further cleaned and re-clustered into 6 different clusters and projected onto a UMAP embedding (Fig. 3B). Freshly isolated cells formed a highly homogenous population; clusters 0, 1, 2, and 3 formed a homogenous subpopulation accounting for over 96% of the total microglia. The smaller clusters 4 (2.4% of cells) and 5 (1.4% of cells) appeared transcriptionally distinct and separate from the rest of the cells in the projection. Using the FindMarkers function, we found that cluster 4 highly expressed markers that are typically seen in activated microglia (*Ccl4, Apoe, Cd74,* and *Ccl12*) and cluster 5 expressed interferon response genes (*Ifit3* and *Ifitm3*), while clusters 0 through 3 had higher expression of homeostatic genes (Fig. 3C). Based on this information, we re-classified these cells as either activated, interferon, or homeostatic (Fig. 3D). Plotting expression of known homeostatic (*Tmem119, Cx3cr1, P2ry12*), activation (*Ccl4, Apoe, Cd74, Ccl12*), and interferon-related (*Ifit3, Irf7*) markers confirmed this classification (Fig. 3E).

To further characterize the molecular signatures of these freshly isolated ex vivo microglia, we performed gene set analysis based on established gene lists from previous studies of microglia activation (Table 2). We tested the three relevant Friedman et al. [4] gene modules (resting microglia, interferon-related, neurodegeneration) and found that both the activated and interferon clusters had lower expression of the resting microglia markers, the activated cluster strongly upregulated the neurodegeneration-related gene set, and the interferon cluster strongly upregulated the interferon-response cluster. Furthermore, the activated microglia cluster had increased expression of genes upregulated in disease-associated microglia [5] and microglia during late response to neurodegenerative disease [31]. Finally, both the activated and the interferon-related cluster increased expression of genes upregulated in injury response microglia seen following focal demyelination from lysolecithin injection [22] (Fig. 3F). Thus, despite originating in a non-transgenic, disease-free mouse, some microglia freshly isolated from the brain still exhibit the activation profile seen in disease models. However, these activated, and interferon-associated cells make up only 3.8% of total microglia and are vastly outnumbered by resting homeostatic microglia.

**Table 2:** The activated microglia cluster exhibits similar transcriptional responses in previous gene sets of activated microglia

Gene Set	Description	Citation	Activated vs Homeostatic Adj_*P*-value	Interferon VS Homeostatic ADJ_*P*-Value
Resting Microglia	Genes marking parenchymal microglia versus other brain myeloid cells	Friedman et al., 2018	6.24E-29	9.39E-03
Neurodegeneration	Genes upregulated in microglia across multiple neurogenerative disease models	Friedman et al., 2018	8.88E-49	7.21E-02
Interferon-related	Interferon-stimulated genes highly induced in response to virus	Friedman et al., 2018	1.00E+ 00	1.65E-15
Keren-Shaul DAM UP	Genes upregulated by disease-associated microglia (DAM) in a 5XFAD model of Amyloidosis	Keren-Shaul et al., 2017	7.93E-23	1.00E+ 00
Keren-Shaul DAM Down	Genes downregulated by DAM in a 5XFAD model of Amyloidosis	Keren-Shaul et al., 2017	9.93E-56	1.71E-05
Mathys Late-respone UP	Genes upregulated in the late-response microglia cluster of a CK-p25 mouse model of neurodegeneration	Mathys et al., 2017	2.17E-23	5.53E-08
Mathys Late-response DOWN	Genes downregulated in the late-response microglia cluster	Mathys et al., 2017	8.46E-74	1.79E-14
Hammond IRM	Genes upregulated by injury response microglia (IRM) in mice in response to a demyelinated lesion	Hammond et al., 2019	9.11E-07	9.24E-06

GENE SET is the name of the gene set used to calculate percentage gene set. For each cell, the percentage of UMIs mapping to genes in each gene set was calculated. DESCRIPTION is a short description of the specific gene set. CITATION points to which published research paper first defined this gene set. ACTIVATED VS HOMEOSTATIC ADJ_*P*-VALUE is the adjusted *p*-value calculated from the two-tailed t-test between percent gene set in the activated cluster against percent gene set in the homeostatic cluster. INTERFERON VS HOMEOSTATIC ADJ_*P*-VALUE lists the adjusted *p*-value calculated from the two-tailed t-test comparing percent gene set in the interferon cluster to percent gene set in the homeostatic cluster. All *P*-values were adjusted for multiple testing using a Bonferroni correction

### Integrated analysis shows that in vitro cells cluster separately from freshly isolated cells

**Fig. 4:**
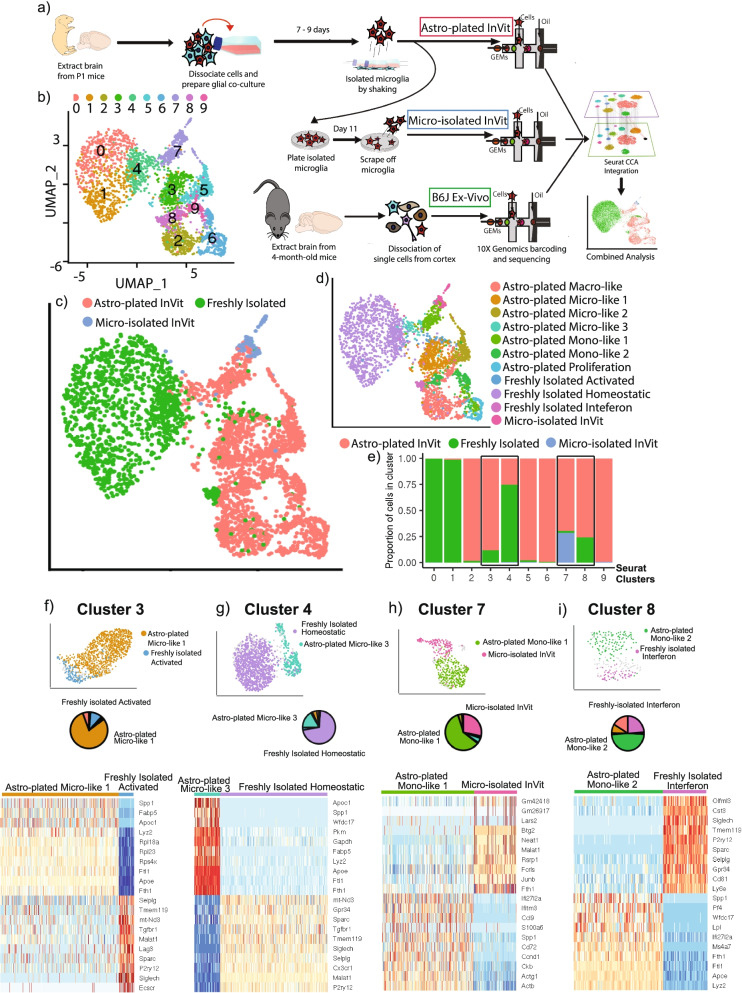
Little overlap between in vitro and freshly isolated microglia transcriptomes. **a** Schematic diagram of microglia isolation and analysis methods. Seurat canonical correlation analysis (CCA) integration was used to perform clustering and UMAP embedding on the combined cell population. **b** Seurat clustered the 13,817 combined cells (8794 freshly isolated cells, 4855 astro-plated in vitro cells, 168 micro-isolated in vitro cells) into 10 populations that show heterogeneity in the UMAP. Cells were downsampled to 2000 for visualization. Astro-plated and micro-isolated cells analyzed in combined UMAP are the same cells analyzed in Fig. 1 and 2. **c** UMAP of the combined cells colored by cell source. Even after CCA integration, distinct separation between microglia from in vitro and freshly isolated sources is apparent. **d** Colored UMAP embedding labeling the cells by previously determined clusters from Fig. 2. **e** Bar graph quantifying the percentage of each Seurat cluster that is made up by cells from the 3 different sources. The majority of clusters are close to 100% represented by cells from a single source, though the 4 highlighted clusters—3,4,7, and 8— originate from mixed sources. **f** Subclustering of the mixed-source cluster 3, highlighting the two largest representative clusters: astro-plated microglia-like cluster 1 and freshly isolated activated cluster. Pie chart shows the proportion of cells in this integrated cluster that are from the different transferred clusters: the two largest components of the cluster are astro-plated micro-like 1 and freshly isolated activated cells, with a minority of cells from other groups including astro-plated macro-like. The 10 top up- and downregulated markers are plotted in a heatmap. **g** The same plots as subpanel **f**, but for integrated cluster 4, highlighting the freshly isolated homeostatic cluster and the in vitro microglia-like 3 cluster. Minority components of the pie chart include astro-plated micro-like 1. **h** The same plots as **f**, but for integrated cluster 7, highlighting astro-plated mono-like cluster 1 and micro-isolated in vitro cells. Minority cell types of the cluster include astro-plated micro-like 3 and astro-plated micro-like 2 cells. **i** The same plots as subpanel **f**, but for cluster 8, which is comprised mostly of the astro-plated monocyte-like 2 cluster and the freshly isolated interferon cluster. Minority cell types of the cluster include astro-plated macro-like and astro-plated micro-like 1 cells

We used Seurat canonical correlation analysis (CCA) to integrate data from the freshly isolated ex vivo, astrocyte-plated in vitro, and microglia-isolated in vitro “monoculture” datasets (Fig. 4A) based on sources of variation common to all three datasets. Microglial cells (*N* = 13,817) were projected on a single UMAP containing ten clusters (Fig. 4B). Clusters 0, 1, and 4 (64.4% of microglia) formed a homogenous supercluster with cells clustered tightly together, whereas the remaining clusters showed more branching and distinct separation, segregating by microglial preparation method (Fig. 4C).

Cell population enrichment analysis identified that the majority of cells from the homogenous supercluster (clusters 0, 1, and 4) originated from freshly isolated homeostatic microglia (Fig. 4D-E). Interestingly, a small proportion of freshly isolated microglia clustered with in vitro astro-plated microglia (clusters 3, 4, 7 and 8, Fig. 4C and E).

To better understand the composition of clusters 3, 4, 7, and 8 that contained both freshly isolated and astro-plated microglia, we subclustered the cells in each of these clusters, determined the proportion of microglial subpopulations in each cluster (pie graphs), and identified genes differentially expressed between the two dominant subpopulations in each cluster (heatmap) (Fig. 4F-I). Cluster 3 was composed predominantly of astro-plated micro-like group 1, followed by a smaller number of freshly isolated activated microglia, indicating that within the in vitro microglia preparations, astro-plated micro-like 1 is most similar to freshly isolated but activated microglia. However, despite the similarities, these microglia still differentially expressed key genes associated with *Tmem119*^*high*^*P2ry12*^*high*^ but *Lyz2*^*low*^*Apoe*^*low*^ homeostatic markers vs *Tmem119*^*low*^*P2ry12*^*low*^ but *Lyz2*^*high*^*Apoe*^*high*^ activation markers (Fig. 4F). This suggests that even the subgroup of in vitro astrocyte-plated microglia that was most similar to activated freshly isolated microglia displayed signs of over-activation. Meanwhile, cluster 4 was composed predominantly of freshly isolated homeostatic microglia, with a smaller percent of astro-plated micro-like 3 microglia. Despite these microglia clustering together, the UMAP of cluster 4 showed that they were spatially separate and mostly non-overlapping subpopulations (Fig. 4G). Furthermore, astro-plated micro-like 3 microglia have low levels of homeostatic markers *Tmem119, Cx3cr1, Sparc*, and *P2ry12*, and high levels of activation markers *Apoe* and *Lyz2*, in comparison to freshly isolated homeostatic microglia, suggesting that even the most homeostatic-like in vitro microglia are more activated than homeostatic freshly isolated microglia. Taken together, these data indicate that in vitro microglia are over-activated in comparison to microglia in vivo, and display increased heterogeneity not observed under normal conditions in the brain.

### The ‘culture shock’ signature of microglia activation in vitro

**Fig. 5:**
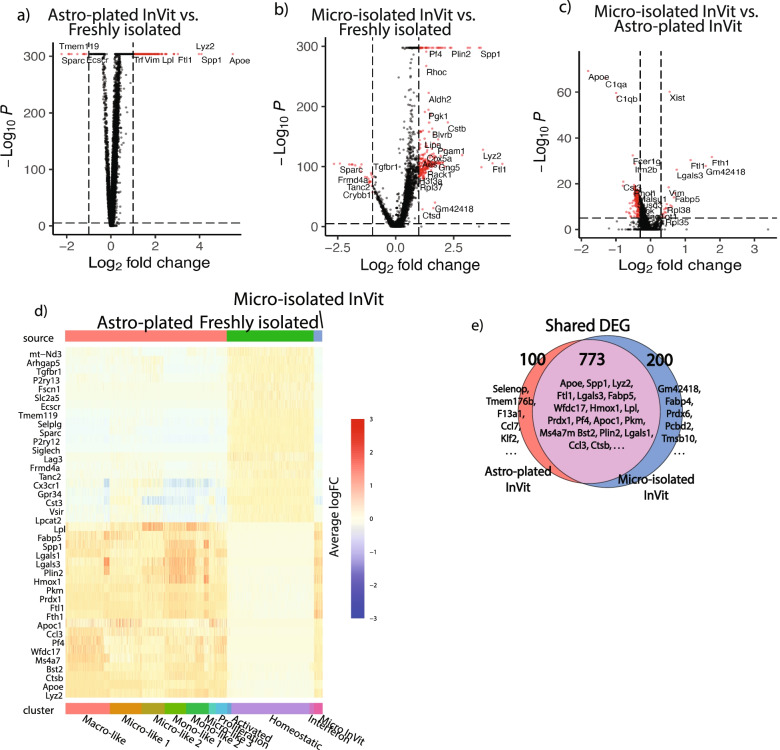
Astrocyte-plated and micro-isolated cultured microglia are marked by a culture shock gene set. **a** Volcano plot of the pseudo-bulk analysis comparing all astro-plated cells to freshly isolated cells. Genes significant with adjusted *p*-value < 0.01 and abs (average logFC) > 1 are highlighted in red. Meeting our strict criteria, we found 9 genes significantly decreased in the cultured microglia and 231 genes significantly increased. **b** Pseudo-bulk analysis of Micro-isolated in vitro cells compared to freshly isolated microglia. Genes with adjusted *p*-value < 0.01 and abs (average logFC) > 1 are highlighted in red. We see similar genes upregulated in this comparison as in the astro-plated in vitro comparison, though at relatively higher *p*-values. **c** Pseudo-bulk differential expression volcano plot of a direct comparison between the two culturing methods, micro-isolated in vitro cells compared to astro-plated in vitro cells. Highlighted in red are genes with adjusted *p*-value < 0.01 and abs(average logFC) > 0.5. **d** Heatmap showing the individual cell expression of the top 20 up- and downregulated genes found in **a.** 2000 downsampled cells are used for visualization purposes. **e** Venn diagram set comparison of the differentially expressed genes (DEG) with abs (average logFC) > 0.5 in the pseudo-bulk micro-isolated versus freshly-isolated microglia, compared astro-plated in vitro versus freshly-isolated microglia

Next, we performed differential expression analysis to investigate the microglial activation signature of cultured microglia compared to freshly isolated microglia. Compared to freshly isolated microglia, astro-plated microglia and micro-isolated monocultured microglia upregulated activation markers such as *Lyz2, Apoe,* and *Lpl* and downregulated homeostatic markers *P2ry12*, *Cx3cr1*, *Sparc,* and *Tmem119* (Fig. 5A-B). When astro-plated cultures were compared to micro-isolated monocultured microglia, we identified many differentially expressed genes, including up-regulation of disease- and activation-associated genes, *Apoe, C1qa*, and *C1qb* in astro-plated cultures, and up-regulation of genes such as *Fth1* and *Hmox1* in micro-plated monocultures (Fig. 5C). Substantial overlap was found in the microglial activation signature under both culture conditions when freshly isolated ex vivo microglia was used as a comparator, with commonalities between micro-isolated monocultures and astro-plated cultures (Fig. 5E). We visualized the top 20 up- and down-regulated genes in the differential expression gene signature of astro-plated and micro-isolated monocultured microglia compared to all freshly isolated microglia (Fig. 5D), highlighting a “culture shock” gene signature of in vitro microglia that was not found in freshly isolated microglia. This signature is characterized by strong upregulation of disease-associated genes including *Apoe*, *Lyz2* and *Spp1*, and downregulation of homeostatic microglial markers, such as *Cx3cr1*, *P2ry12*, and *Tmem119*.

### Predictive network modeling revealed potential key drivers of the in vitro microglial phenotype

In addition to finding culture shock genes, we sought to understand how gene networks are disrupted in cultured microglia. We applied MEGENA to generate gene networks for freshly isolated (Fig. S1) and astro-plated (Fig. S2) microglia. The gene hubs of these inferred networks recapitulated the top differentially expressed genes across microglial preparations, strengthening our findings that cultured microglia are highly transcriptionally dissimilar to microglia in the brain. However, because MEGENA uses a co-expression rather than causal network analysis approach, it cannot identify potential upstream master regulators (key drivers) for microglial states in vitro. Similarly, the genes that are most differentially expressed between cultured and freshly isolated preparations are not necessarily the same genes that drive the cultured phenotype. For example, a gene responsible for regulating many others downstream may only need to be slightly differentially regulated in culture to produce an outsized effect on the transcriptome of microglia. We therefore employed a predictive network model [13, 15, 16] to construct causal genetic regulatory networks that might regulate microglial state transitions in culture.

**Fig. 6:**
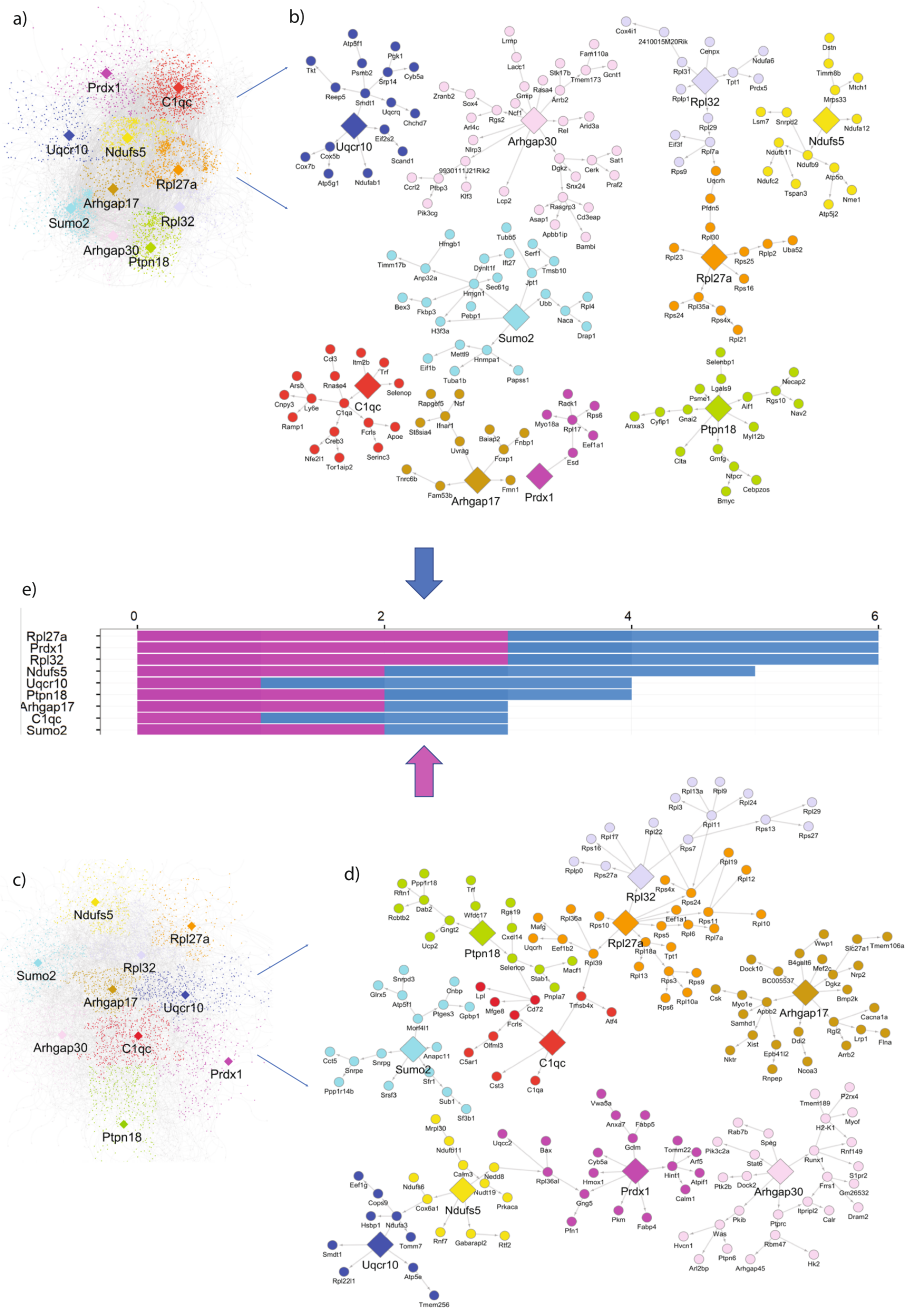
Causal predictive network modeling reveals potential key drivers of in vitro microglia state. **a** Causal genetic regulatory network of astro-plated microglia with top inferred 10 key drivers *Rpl27a, Prdx1, Rpl32, Ndufs5, Uqcr10, Ptpn18, Arhgap17, C1qc, Sumo2, Arhgap30*) enclosed in diamonds and (**b**) Subnetwork of **a** showing downstream effector genes; (**c**) Causal genetic regulatory network of micro-isolated microglia and (**d**) its subnetwork. **e** The top 9 (out of total 444) key drivers replicated across both microglial cohorts (astro-plated and micro-isolated microglia) ranked by prioritization score, with larger scores indicative of greater confidence in a key driver as a modulator of microglial activation

We generated two causal networks for the astro-plated (Fig. 6A) and micro-isolated/microglial monoculture (Fig. 6C) preparations. 3847 genes were common to both network gene sets (77% of astro-plated and micro-isolated, *p*- < 2.2e-16), indicating the reproducibility of network analyses across the two culture conditions. We next applied Key Driver Analysis (KDA) [17] to identify upstream master regulators in the causal network that may modulate downstream effector genes. These key drivers are shown enclosed in diamonds. Both cultured microglial networks are driven by the same ten key drivers, including immune and microglial gene *C1qc*. We also identified downstream effector genes that are modulated by the key drivers in the astro-plated and micro-isolated networks. The top nine key drivers of the cultured microglia networks are shown in Fig. 6C (the full list of 444 key drivers is available in Fig. S3). Key drivers were ranked according to the following parameters: whether a key driver gene is present in both datasets, the number of categories of downstream effector genes that nominate the gene as a key driver, and the number of total downstream effector genes that nominate the gene as a key driver.

To better understand the drivers of the cultured phenotype, we plotted the subnetworks of the top key drivers *(Rpl27a, Prdx1, Rpl32, Ndufs5, Uqcr10, Ptpn18, Arhgap17, C1qc, Sumo2*, and *Arhgap30*) in astro-plated (Fig. 6B) and micro-isolated microglia (Fig. 6D). Notably, downstream effector genes in these subnetworks include the disease genes *Apoe* and *Lpl*, which are influenced by key driver *C1qc* in the astro-plated and micro-isolated networks, respectively.

### Knockdown of C1qc decreases activation of cultured microglia

To test the predictions from our casual network analysis, we selected two top targets, *C1qc* and *Prdx1*, to validate by siRNA knockdown in vitro. Of the top ten targets, eight (*Prdx1, C1qc, Uqcr10, Ndufs5, Rpl27a, Sumo2, Ptpn18, and Rpl132*) were differentially expressed between cultured and fresh preparations. Of these eight, *Prdx1* was the most upregulated in culture (3.3 times higher in culture, with expression in 100% of cultured cells and only 56% of freshly isolated cells), while *C1qc* was the least upregulated (1.03 times higher in culture, with expression in 99.5% of cultured cells and 100% of freshly isolated cells). Thus, these two targets represent two different types of potential key drivers: genes that are highly differentially expressed across conditions (*Prdx1)* and those that are differentially expressed to a much lesser extent (*C1qc*) but may nonetheless exert disproportionate influence on the transcriptome.

**Fig. 7:**
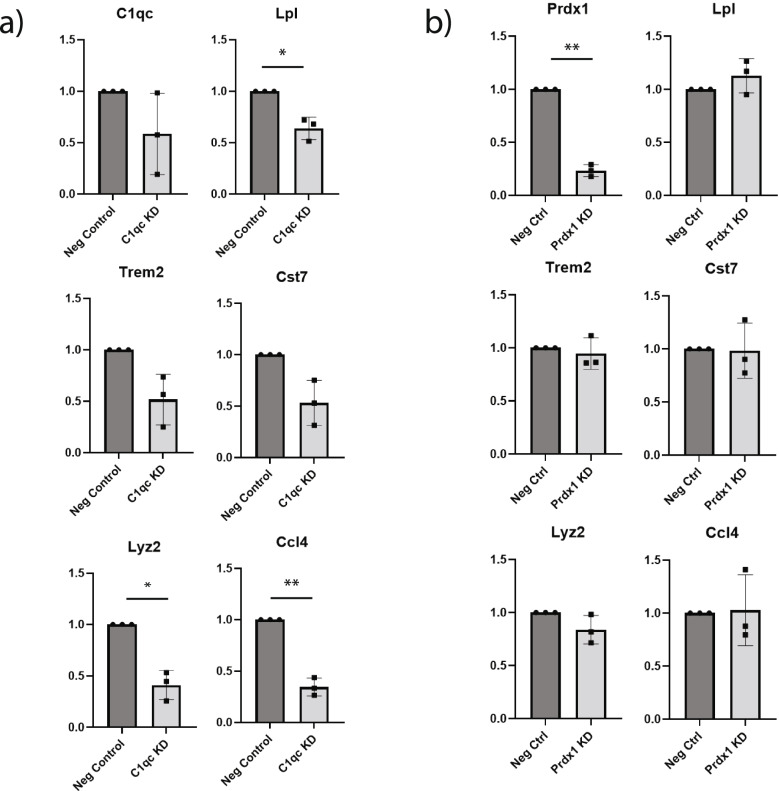
Knockdown of predicted key driver C1qc, but not Prdx1, dampens microglial activation in vitro. Expression of activated microglial genes *Lpl, Trem2, Lyz2, Cst7*, and *Ccl4* following knockdown of (**a**) *C1qc* or (**b**) *Prdx1*. Expression is presented as fold change, normalized to GAPDH and negative control. *n* = 3 mice per group, two-tailed paired t test; *, *p* < 0.05; **, *p* < 0.01

We knocked down *C1qc* and *Prdx1* in a mixed glial co-culture and performed RT-qPCR to quantify transcript levels of activated microglial genes *Lpl*, *Trem2*, *Lyz2*, *Cst7*, and *Ccl4* (Fig. 7). Despite variable knockdown efficiency of *C1qc* we observed significant reduction in expression levels of microglial activation genes *Lpl*, *Lyz2*, and *Ccl4*, while *Trem2* and *Cst7* showed a trend toward downregulation (Fig. 7A). Notably, *Lpl* is one of the genes predicted by the micro-isolated causal network to be regulated by *C1qc.* Conversely, *Prdx1* knockdown was robust, but did not significantly affect expression of activation genes (Fig. 7B). Our knockdown results confirmed that genes with small differential expression fold-changes may exert significant effects, highlighting the importance of key driver discovery for network modeling.

## Discussion

In vitro culture systems are a popular method of studying microglia and characterizing their phenotypes, functions, and responses to insults or other stimuli. In the present study, we used scRNAseq to characterize the transcriptional phenotypes of freshly isolated microglia and two in vitro preparations of microglia: microglia co-cultured with astrocytes (astro-plated) and grown in monoculture (micro-isolated). We found that microglia in vitro are marked by widespread activation and are highly heterogenous, consisting of multiple transcriptionally distinct subpopulations; a subset of these were not microglial (monocyte-like, macrophage-like), while others were characteristic of microglia in activated or other non-homeostatic conditions (neurodegeneration, interferon response, LPS), despite the absence of any insult in culture. We identified a unique ‘culture shock’ gene set that defined the widespread activated phenotype in culture; this gene set was dominated by upregulated disease genes such as *Apoe*, *Lyz2*, and *Lpl*, and downregulated homeostatic genes, such as *Tmem119*, *P2ry12*, and *Cx3cr1*. In contrast, freshly isolated microglia were highly homogenous, and consisted predominantly of homeostatic or resting microglia, with a small number of activated or interferon-associated cells.

Importantly, even the least activated and most-microglia like subpopulations from the in vitro astro-plated preparation are significantly more activated than both homeostatic and activated freshly isolated microglia. In other words, cultured microglia are not merely activated in the absence of stimuli, they are overactivated, and the phenotype of microglial activation in culture and microglial activation in vivo are dissimilar. This overactivation is particularly problematic for studies of neurodegenerative disease or other insults – such as viral infection, LPS, or other inflammatory challenges – that result in microglial activation, as the baseline activation in culture makes it difficult to distinguish between activation caused by culture conditions and activation caused by experimental challenge. Subtle effects caused by experimental challenge may be masked by the basal overactivation caused by culture conditions.

A common motivation for the use of microglial cultures is the desire to study microglia alone, without the influence of other cell types in the brain (for example, in studies of cell-autonomous signaling). However, our data showed that cultured microglia consist of disparate subtypes, whose vastly different transcriptional profiles suggest a non-uniform response to experimental challenges; the heterogeneity of microglia in culture suggests a heterogeneity in response type and intensity. Importantly, this heterogeneity appears to be an artifact of culture and loss of CNS signaling, and not a biological effect of microglia having been extracted from the brain at an early developmental timepoint. Although early microglia are known to be more heterogeneous, and a transitional cell type that expresses genes associated with both microglia and brain border macrophages does exist in the brain at E14.5 [22], this does not explain the heterogeneity observed in culture for two reasons: first, cultured microglia in our study were harvested at an early postnatal stage, past the developmental window when heterogeneous microglia/brain border macrophage cells are observed, and second, the genes that characterize mature resting microglia and brain border macrophages are not co-expressed at high levels in the same subpopulations of cells. This suggests that the heterogeneity of microglia in culture does not recapitulate that of the brain early in development.

Because microglia require signaling from neighboring cells to maintain their homeostatic phenotype, loss of this resting state and increase in activation is an inevitable problem across all in vitro systems. For instance, the binding of neuronal CD200 to its microglial receptor CD200R is necessary for restricting microglial activation in disease models including rotenone neurotoxicity [32] and *Toxoplasma* encephalitis [33] in the absence of this interaction, microglia become reactive and excessively proliferative. Furthermore, transcriptomic profiling of ex vivo microglia shows a rapid loss of homeostatic gene signatures within hours of removal from the CNS, though this phenotype may be partially rescued by engraftment back into the brain [34]. A recent study [35] demonstrated that this homeostatic phenotype is at least partly regulated by TGF-β2 signaling from astrocytes and neurons, supporting an earlier finding that the addition of TGF-β to culture aids microglia in developing a transcriptional signature that is somewhat more similar to what is observed in vivo [8].

Numerous attempts have been made to reduce activation in culture, including co-culturing microglia with other CNS cells, and culturing cells serum-free. We have demonstrated that even co-culture of microglia with astrocytes is inadequate for maintaining microglia in a homeostatic state in vitro. Intriguingly, we observed that microglia removed from astrocyte co-culture and grown in monoculture for three days before sequencing downregulate activation genes including *Apoe*, *C1qa*, and *C1qb* (Fig. 5C) compared to microglia co-cultured with astrocytes, though this analysis is limited by the comparatively small number of microglia from monoculture captured during sequencing. Baxter et al. [35] recently demonstrated that co-culture with both neurons and astrocytes reduces microglial activation compared to monoculture, and co-culture with only neurons or only astrocytes, but microglia in this triple culture system have not been compared directly to freshly isolated microglia, so whether this system recapitulates in vivo phenotype is unknown. Similarly, microglia cultured in serum-free conditions display reduced but still significant activation [34], suggesting that the presence of serum in culture alone cannot explain the activation we and others have observed in vitro.

Although it is clear that the absence of native CNS signaling in culture results in alteration of the microglial transcriptome, it is not known which specific genes drive these changes. We found that knockdown of *C1qc*, a gene predicted by our causal network model to drive the culture shock phenotype, results in downregulation of microglial activation genes. Notably, this effect occurred despite *C1qc* being only weakly differentially expressed between cultured and ex vivo microglia and despite the fact that we achieved only a modest knockdown of *C1qc* itself. This suggests that even genes that are only minimally differentially expressed may still play a key role in modifying the overall transcriptional phenotype of the cell. Conversely, the lack of effect of knockdown of *Prdx1*, a highly differentially expressed gene, suggests that a high degree of differential expression does not guarantee a gene is an upstream driver of a particular phenotype. Thus, while causal network analysis is a valuable tool for identifying “unlikely” key driver candidates that may be missed by traditional differential expression analysis, validation of these targets remains necessary for parsing out biological significance.

## Conclusions

Our results support the previous body of work demonstrating that the removal of microglia from the brain alters their phenotype, skewing it toward a highly activated profile. Additionally, we have found that cultured microglia consist of disparate subpopulations marked by different molecular signatures characteristic of microglia in injury and disease, and that the gene networks of these populations are radically altered compared to ex vivo microglia. Using causal network analysis, we have identified *Prdx1*, *Ndufs5*, *C1qc*, and *Ptpn18*, among others, as possible drivers of the activated phenotype in culture. By knocking down *C1qc* in culture, we have demonstrated that targeting even weakly differentially expressed genes may serve as a viable approach for modulating microglial activation in vitro.

## Supplementary Information

**Additional file 1: Table S1.** Genes differentially expressed in each cluster of primary cultured astrocyte-plated microglial cells. All genes displayed have an adjusted *p* value of less than 0.05 and are expressed in a minimum of 25% of cells in each cluster.

**Additional file 2: Figure S1.** MEGENA network analysis of freshly isolated cells establishes a reference transcriptional network for microglia. **(a)** Transcriptional regulatory network for freshly isolated microglia inferred by MEGENA. Nodes represent genes, and an edge between nodes represents the significant correlation of those two genes. Bar graph shows relative strength of these top 20 nodes and highlights the module to which each gene belongs. Heatmap shows the scaled expression of the top 100 genes of each of the 3 core MEGENA modules. Heatmap is represented by a downsampled 2000 of the freshly isolated cells. **(b)** Subnetwork of module 2. Weighted module expression is calculated by calculating the sum of the expression of each gene in the module, weighted by its strength in the network, and signed by whether that gene has positive or negative correlation with the largest hub of that module. Plotting the module weighted sum in a violin and feature plot shows strong, significant association of module 2 with the activated cluster (two-tailed t-test between activated vs homeostatic cluster, *p*-value = 1.93e-15). Metascape gene ontology (GO) enrichment suggests that this module is related to inflammatory response and the transcriptional response to stress. **(c)** Subnetwork of module 3, containing many homeostatic microglia markers. Weighted module expression is highest in the homeostatic cluster and significantly decreases in cells from the activated cluster (two-tailed t-test between activated vs homeostatic cluster, *p*-value = 4.34e-60). Metascape shows that pathways enriched for this module include ribonucleoprotein complex formation, functioning of the lysosome, and immune pathways like neutrophil degranulation and myeloid activation. **(d)** Network of the subnetwork for module 4, which contains many interferon-related genes. Module weighted sum is significantly increased in the interferon cluster compared to the homeostatic cluster (two-tailed t-test, *p*-value = 7.65e-23). Metascape enrichment analysis shows pathways related to interferon signaling and cytokine production.

**Additional file 3: Figure S2.** In vitro microglia networks are disrupted and dissimilar to the freshly isolated network. **(a)** Transcriptional regulatory network for astrocyte-plated in vitro cells inferred by MEGENA. Top 50 nodes are labelled, and nodes are colored corresponding to the 5 core modules identified by MEGENA clustering analysis. **(b)** Bar graph of the top 40 nodes shows from **a** relative strength between nodes. **(c)** Heatmap of the scaled expression of the top 75 genes of each core module across 2000 downsampled cells. Modules strongly associate with specific clusters. Module 9 contains genes highly expressed in the macrophage-like cluster. Module 5 contains genes associated with the microglia-like 1, and monocyte-like 1 and 2 clusters. Module 6 contains hubs of ribosomal genes and is downregulated in microglia-like cluster 2 and upregulated in microglia-like cluster 3. Modules 7 and 8 contain cell cycle genes associated with proliferation cluster. **(d)** Subnetwork of module 5, a heterogenous module that contains a variety of hubs. Hubs like *Cx3cr1*, *Fcrls*, and *Fcer1g* are markers of resting microglia, while hubs like *Apoe, Lgals1, Lgals3* are activation markers. Smaller hubs like *Anxa1* and *S100a4* are unique to monocyte-like cells. The weighted module expression of this module is strongest in the microglia-like 1 cluster, but expression of this module is robust across all clusters. Metascape GO enrichment shows enrichment for immune and cell death processes. **(e)** Subnetwork of module 7, a cell cycle module whose module weighted expression is significantly higher in proliferation cluster 6 (two-tailed t-test, *p*-value = 1.62e-166). Metascape analysis of this clusters shows enrichment of cell cycle processes. **(f)** Subnetwork of module 9, with hubs *Mrc1* and *Stab1* that are unique markers of macrophages. Weighted module expression of this module is expressed robustly in all clusters, but is highest in macrophage-like cluster 0 (two-tailed t-test, *p*-value = 8.23e-7). Metascape GO enrichment shows this module is related to lysosomal and vesicle transport pathways.

**Additional file 4: Figure S3.** Key drivers of the cultured microglia phenotype. Top 100 of 444 total key drivers with prioritized rank derived from the astro-plated microglia and micro-isolated microglia networks.

## Data Availability

The datasets used and/or analyzed during the current study are available from the corresponding author on reasonable request.

## Abbreviations

AD: Alzheimer’s disease
ALS/FTD: Amyotrophic lateral sclerosis/frontotemporal dementia
CCA: Canonical correlation analysis
DE: Differentially expressed
GO: Gene ontology
KDA: Key Driver Analysis
MEGENA: Multiscale Embedded Gene Co-expression Network Analysis
MS: Multiple sclerosis
PCA: Principal component analysis
PD: Parkinson’s disease
scRNAseq: Single-cell RNA sequencing
UMAP: Uniform manifold approximation and projection
