# Supplementary material for: Culture shock: microglial heterogeneity, activation, and disrupted single-cell microglial networks in vitro
Source: Mol Neurodegener. 2022 Mar 28;17:26. doi: 10.1186/s13024-022-00531-1 (PMC8962153; doi:10.1186/s13024-022-00531-1)
Supplement: Supplementary file 1 — Additional file 1: Table S1. Genes differentially expressed in each cluster of primary cultured astrocyte-plated microglial cells. All genes displayed have an adjusted p value of less than 0.05 and are expressed in a minimum of 25% of cells in each cluster. [file 13024_2022_531_MOESM1_ESM.docx]

**Table S1**

| **CLUSTER** | **Upregulated genes** | **Downregulated genes** |
| --- | --- | --- |
| 0 | Mrc1, Cd38, Fcgr2b, Ms4a7, Stab1, Gpx3, Bst2, Maf, Cd28, Wwp1, Wfdc17, Lrp6, Selenop, Grn, Rapsn, Cnrip1, Gas6, Aoah, Hpse, Ednrb, Cela1, Ang, Ltc4s, Fcna, Clec4n, Paox, Itsn1, Lamp1, F630028O10Rik, Iqgap2, Pf4, Ier3, Tnf, Gpnmb, Npl, Ms4a6c, Gm26522, Wwp2, Hexa, Rnasel, Arhgap19, Tbc1d4, Sptssa, Bcl2a1b, Gramd1b, Cd68, Clta, Trf, Snx6, Clcn5, Ninj1, Reps2, Ccl7, Prps2, Fam213b, Ifi27, Ccr1, Slc9a9, Prkacb, Fcgrt, Cd93, Rnase4, Dab2, Idh2, B2m, Fam213a, St6gal1, Sgpl1, Ctsb, Kif3a, Mgst1, C1qc, Ulk2, Epb41l3, Bmp2, Abcg3, Snx3, Chchd10, Rnf141, Stard8, 0610012G03Rik, Cpq, Eps15, Ap1b1, Ctsa, Cp, Mtss1, Cd164, H2-K1, Slc43a2, F13a1, Tpp1, Lyz2, Abca1, Eps8, Slc26a11, Pikfyve, Gns, Ap2a2, Pnpla7, Ttyh3, Serpinb6a, Mmp27, Pla2g7, Nfkbid, Gsta4, Galc, Herc1, Rgl1, Snx2, Pltp, 1700003F12Rik, Slfn5, Gm1673, Fcrls, Nrp1, Kif13b, Lbp, Gm5617, Fkbp15, Tmem106a, Adam9, mt-Co1, Ccl3, Mfsd1, Dse, Mndal, Ddx60, Rnf213, Wdfy3, Igfbp4, Smagp, Zdhhc14, Gtf2i, Lgals3bp, Ifi203, Dmxl1, Arhgap15, C1qa, Myo5a, Erbin, Marcksl1, Ms4a6b, Raph1, Sgpp1, Cryba4, Ttc3, AC149090.1, Aph1c, Gstm5, Itgav, Mdfi, P2rx4, Fosb, Tmem256, Amdhd2, Cd84, Rap2b, Cd33, Sec14l1, Crybb1, Apoc4, Palld, Aplp2, Frmd4b, Mif, Blvra, Gapvd1, Timp2, Fgd4, Cxcr4, Npc1, Mpp1, Man2a1, Il4ra, Sbf2, Adgre1, Ap2s1, Man1a, Etv1, Mdfic, Tln2, Fndc3a, Scly, Zfp704, Tnks2, Mroh1, 2610507B11Rik, Cltc, Bbx, Fchsd2, Plxnb2, Myo7a, Rab31, Eif4g3, Naglu, Atp6v0a1, Appl2, Sash1, Cd200r4, Egr2, Dbp, Scn1b, Arap2, Tmem87b, Tbc1d12, Smim1, Ppp1r9a, Apoe, Peli1, Man1c1, Ramp1, Gaa, Hacd4, Slc17a5, Dcxr, Pla2g16, Acat1, Mpst, Plxnc1, Tnfaip2, Nme4, Cpne3, Lgals9, Gyg, Zbtb38, Gnpda1, Ptbp3, Plod1, Secisbp2l, Vegfb, Fcrl1, S100a1, Slc31a1, P4ha1, Selenbp1, Hpgds, Lpar6, Rbpj, Tecpr1, Lrp1, Tagap, Man2b2, Stat1, Scd2, Phyh, Twf1, Fcho2, Ccrl2, Ncoa4, Cited2, Gadd45b, Parp14, Rtp4, Vegfa, Zcchc6, Idh1, Trappc6a, Nrp2, Ccl2, Msrb1, Ly6e, Osbpl9, Sox4, Ankrd37, Plek, Pmepa1, Fn1, Trim30a, Isg15, Srgn, Gmfg, Rgs1, Hgsnat | Cd72, Lpl, Cd52, Ywhah, Capg, Lat2, Coro1a, Cd81, Basp1, Fth1, Taldo1, Slc11a1, Ckb, Cd34, Selplg, Serpine2, Clic1, Rhoa, Lsp1, Cd74, Tmsb4x, Lair1, Apbb1ip, Alox5ap, Olfml3, Rhob, Cst7, Irf5, Itgb2, Mpc1, Mcub, Glipr1, Nes, Sparc, Plxdc2, Gnas, Chst1, Slamf9, Csf2ra, Eif1, Actr3, Cd9, Adam8, Cd14, Alcam, Emp3, Gngt2, Sgk1, Tspo, Cdkn1a, Ctss, St3gal6, Cebpa, Itgb5, Abi3, Atf3, Ctsl, Rtn4, Pdgfb, Pea15a, Actb, Myo1e, Spi1, Cap1, F11r, Plp2, Ubc, Tpm4, Smim3, Hexb, Bin2, Tmem176a, Zfp36l2, Igf1, Tmsb10, Crip1, Fxyd5, Vim, Jund, Plaur, C5ar1, Spp1, Esd, Cd300lb, Ccl9, Ppfia4, Mafb, Rnh1, Tubb4b, Tmem176b, Lpcat2, Ldhb, Actg1, Asph, Hmox1, Ccl6, Glrx, Hpgd, Tubb2a, Tubb2b, Btg1, Aprt, Ptpre, Cst3, Lgals3, Tmem119, Lrrfip1, Tuba1c, Cebpb, Asns, Gadd45g, Rab7b, Cdkn2a, Phgdh, Gclm, Bbc3, Ier5, Gpr84, 2010111I01Rik, Smad7, Glul, Mthfd2, Tagln2, Plin2, Rassf1, H3f3b, Tubb5, Gpr34, Anxa2, Pgd, Sqstm1, Btg2, Irf8, Stap1, Irf2bpl, Cenpa, Tuba1a, Ifi27l2a, Rhoc, Fgfr1op, Srxn1, Ube2s, Cks2 |
| 1 | Cst3, Cd81, Tmem176a, Cd34, Ctss, Olfml3, Tmem176b, Serpine2, Cst7, Ldhb, Ctsh, Sparc, Gpr34, Itgb5, Ctsl, Rgs10, Laptm5, Rpl21, Gngt2, Rpl7, Cd74, Cd52, Hexb, Mpc1, Plxdc2, Fau, Apoe, Vsir, Rplp0, Glul, Lpcat2, Abi3, Ly86, Rps4x, Lsp1, Selplg, Rpl18a, Apoc1, Jam2, Rpl35a, Lag3, F11r, Cd9, Ssr4, Btg2, H2-DMa, Rps9, Sgk1, Gm2a, Fcgr3, Smad7, Slc11a1, Tmem37, Cx3cr1, Tyrobp, Creg1, Man2b1, Ppfia4, Tmem119, Egln3, Csf1r, Itm2c, Ctsz, P2ry12, Fgfr1op, Rps27, Fam46c, Chst1, H2-Ab1, Itgam, Rnaset2a, Cebpa, Ifngr1, Cd300c2, Cd37, Socs3, Lat2, Ypel3, Junb, Susd3, Ptms, Btg1, Aif1, Frmd4a, Rhoh, Golm1, Cxxc5, Ptpre, Arl5c, Dennd4a, Kctd12, Pros1, Cmtm6, Atp8a1, Fcrls, Dnajb14, Ank, Zfp36, Sdf2l1, Ccl6, Bnip3, Fabp5 | Ms4a7, Mrc1, Cd38, Serpinb6a, Lgals1, Ifitm3, Bst2, Dab2, Fcna, Snx2, Igfbp4, Iqgap2, Wfdc17, Cfp, Prdx1, Ehd1, Iqgap1, Gpnmb, Wwp1, Cd93, Cela1, Clec12a, Plin2, Tmem106a, Arpc3, Mndal, Pf4, Metrnl, 0610012G03Rik, Hist1h2bc, Clec4d, Itsn1, Idh2, Ccnd1, Eea1, Cenpb, Snx6, Akr1a1, Ifi203, Sh3bgrl, Eif2s2, Cox5a, F630028O10Rik, Snx3, Txnip, Lipa, App, Folr2, Ccl2, H2afj, Eid1, Hip1, Vav3, Ednrb, Aoah, Slfn2, Gpx3, Cd36, Adgre1, Tagln2, Stab1, Gyg, Rps27l, Rhoc, Cox7a2, Ifi207, Lyz2, Ap2m1, Rtp4, Uqcr10, Mpp1, Kif3a, Arhgap10, Osbpl8, Ccl7, Cd28, Bmp2, Atp6v1e1, Actg1, Palld, Rbpj, Lilr4b, Anxa1, Ap2s1, Ninj1, Unc119, Cstb, Isg15, Cybb, Ctsb, Hint1, 1810058I24Rik, Atp5b, Spp1, Hmox2, Mtss1, Ndufc1, Ifi211, Sf3b5, H2-D1, Aldh2, Maf, Hist1h1c, Anxa5, Nop10, Tnf, Scand1, Ndufab1, Txn1, Ifitm2, Uqcrq, Rtn4, Mgst1, Snx8, Blvra, Bcl2a1b, Ndufa4, H2-K1, Tpr, Rap2b, Npl, Ube2s, Nagk, Actr3, Oasl2, Clec4n, Smdt1, Sptssa, Calm1, Gsn, Mrpl57, Hk3, Kpna4, Ddit3, Vim, Smpdl3a, Hspa9, Gdpd1, B4galt6, Pla2g16, Myl12a, Bax, Atp6v0a1, Nrros, Reps2, Stom, Jun, Sars, Smc4, Pcbd2, Dnajc15, Prkacb, Msr1, Ccr1, Rnasel, Hpse, Lmna, Psmd2, Stmn1, Fam213b, Rnf141, Slc40a1, Pfn1, Qpct, Bex3, Nrp1, Atp6v1a, Cfdp1, Asns, Stat1, Ran, Oas1a, Ms4a6c, Mcm6, Cd200r1, Vat1, Arhgap15, Amdhd2, Wwp2, Eps15, Ap2a2, Smagp, Fcgrt, Hnrnpab, Pltp, Mthfd2, BC005537, Arhgap19, Stap1, Ifit3, Rpl22l1, 2610001J05Rik, Tubb5, Nap1l1, Gmnn, Stard8, Ssrp1, Rnf213, Msrb1, Gars, Marcksl1, Sec14l1, Eif3a, H1f0, H2afv, H3f3b, Hgsnat, Plk2, Agfg1, Sae1, Cdkn1a, Srsf7, Adam8, Ghitm, Plau, Mdfic, Snrpd1, Speg, Nrp2, Clic4, Pcna, Nop58, Ifi27, Cyb5r3, Tubb4b, Cdk6, Cks1b, Sp100, Psmd8, Atf4, Dnmt1, Por, Hmox1, Gas6, Dok2, Paox, Dut, Ctsc, Lgals3bp, Smc2, Rnh1, Lrp6, Tbrg1, Tyms, Slfn5, Ifi27l2a, Cited2, Rapsn, Cycs, Lig1, Cnrip1, Pid1, Anp32b, Ranbp1, Cdk1, Esd, Irf7, Lgals3, Dnajc9, Malat1, Dtymk, Ccl12, Hmgb2, Phf11d, Fam111a, Card19, C5ar1, Pmp22, Ifrd1, Rrm1, Il4ra, Tmpo, Hat1, Nucks1, Hist1h1e, Nupr1, Anxa2, Cd72, Tuba1c, Hmgb1, H2afx, Srxn1, Flna, Glipr1, Incenp, Mdm2, Rgs1, Gm26522, Neat1, Cks2, Tubb2a, 2010111I01Rik, Fcgr2b, Ang, Tuba1b, Jund, Atad2, Bbc3, Sqstm1, Hmgn2, Ccl9 |
| 2 | Rassf1, H3f3b, Klf6, Ubc, Rbm39, Bbc3, Atf4, Ier5, Malat1, Trib3, Mylip, Son, Cebpg, Rhob, Irf5, Eif5, Pdgfb, Lpl, Cebpb, Ddx5, Irf8, Slc3a2, Ddit3, Zfp36l2, Ifrd1, Gtpbp2, Rsrp1, Mafg, Snx18, Pafah1b1, Zbtb7a, Ifngr2, Rrbp1, Mcl1, Smim3, Tmem55b, Pnrc1, Basp1, Hnrnpk, Sqstm1, Spi1, Cdk9, Plekho1, Btg1, Atf3, Cebpa, Jun, Pou2f2, Prkcd, Cdkn1a, Eif3a, Slc11a1, Bcl10, Jund, Sowahc, Klf13, Mtdh, Srrm2, Zfand5, Rap1a, Sgk1, Lair1, Bhlhe41, Nktr, Kmt2e, Csnk1e, Cnbp, F11r, Ube2d3, Rab14, Mef2a, Lrrfip1, Mafb, Arhgap45, Zeb2, Arl4c, Zfp91, Acbd3, Hist1h1c, Yy1, Hnrnpa0, Srsf11, Irf2, Xbp1, Pura, Gpr84, Ccnl1, Coro1a, Mthfd2, Purb, Kpna4, Clk1, Lemd2, Arpc5l, Abcg1, Ptprc, Eif4a1, Hspa9, Lims1, Kcmf1, Wasf2, Txnip, Wsb1, Dusp11, Retreg1, Ptchd1, Hnrnpf, Nmt1, Irf2bpl, Pgd, Tmem11, Brd2, Mef2c, Rbm25, Kif5b, Tgif1, Itga5, Eif1, Pcbp1, Samsn1, Tgfbr1, Tra2b, Ppig, Dok1, Orai1, Brd1, Arrb2, Rsrc2, Psmd7, Ubxn4, Luc7l2, Tln1, Skil, Irf2bp2, Arf4, Eprs, Csf2ra, Psmd11, Pnn, Gsk3b, Mycbp2, Mdm2, Tifa, Hnrnpdl, Nfkbia, Slc7a5, Relb, Nrbf2, Nupr1, Cd14, Cyth4, Snrnp70, Eif1a, Abi3, Arglu1, Krcc1, Junb, Eif3c, Neat1, Ankrd11, Srrm1, Srsf10, Sars, Chst1, Adap2, Mid1ip1, Mob3c, Apobec1, Nfil3, Iqgap1, Clk4, Actr3, Mgat4b, Nfe2l2, Lmo4, Arl8a, Pnisr, Cd9, Mrpl43, Arhgap25, Tubb2a, Ywhah, Chic2, Zfhx3, Ktn1, Slc39a7, Otud1, Arhgap5, Ggnbp2, Cttnbp2nl, Cyfip1, Bin2, Il10ra, Il6st, Ddx50, Sfr1, Plin2, Fam212a, Ppp2ca, Atxn7l3b, Cd72, Rgs2, Sbds, Tlr2, Ptpn6, Rnf149, Fgf13, Akirin2, Neurl3, Runx1, Csnk1a1, Smap2, Glipr1, Cggbp1, Vav1, Sf3b1, C3ar1, Capza2, Tnfaip8l2, Fyb, Nfic, Psmd12, Tcf25, Mob4, Nab2, Snhg12, Copb1, Chd7, Nars, Vps37b, Nuak1, Hist1h1e, Clint1, Ptpn1, Fus, Srgap2, Mfsd12, Prpf38b, Srsf2, Gnb2, Rheb, Senp6, Ifngr1, Bcl6, Hbp1, Snx20, 5430416N02Rik, Ttc14, Adrm1, Marcks, Ch25h, Hlx, Otulin, Sfpq, Apbb2, Tuba1a, Ptpre, Slc16a3, Eif5b, Bin1, Eif2s2, Oser1, Gtf2h2, Irak2, Ncf1, Tbpl1, Utp3, Nipbl, Taf15, Hcar2, Tax1bp1, Pdgfa, Cx3cr1, Lcp2, Psmc6, Tmem189, Apbb1ip, Derl1, Smap1, Arhgap30, Fam96b, 1700017B05Rik, Rtn4, Ppp1r15a, Zfp622, Srsf5, Lyl1, Rassf3, Mier1, Smg9, 2810474O19Rik, Gpr183, Luc7l3, Sntb2, Ddx6, AU020206, Vasp, Set, Icam1, Nrbp1, Arf1, Fmnl1, Klf4, Eif2s1, Traf2, Ssh2, Eif6, Vapa, Dnttip2, Btg2, Mapkapk2, Riok3, Capg, Wdr26, March7, Lilrb4a, Ppp1r12c, Ube2f, Trappc6b, Fam104a, Bmp2k, Bax, Tnip2, Rgs3, Hspa5, Gatad2b, Fermt3, Ing4, Cited2, Hexb, Mbnl2, Tra2a, Rmi1, Spag9, Rala, Tcirg1, Ube2b, Mbnl1, Cflar, Fam49b, Gtpbp4, Gadd45g, Tmed5, Serinc1, Asph, Ptger4, Ppp2r2a, Tmed2, Twf2, BC017643, Acin1, Hhex, Rsl1d1, Ccnl2, Slc38a2, Bclaf1, Txnl1, N4bp1, Ndel1, Erf, Tspan14, Puf60, Lat2, Mat2a, Matr3, Cmip, Arf6, Tfe3, Morc3, Tnfrsf1a, P2ry6, Ube2d2a, Eif4h, Maz, U2af1, Slc25a25, Arhgap22, Stx5a, Rbm7, Tmbim1, Csnk2a2, Gna15, Srsf7, Gpsm3, 1810032O08Rik, Cfl2, Klf3, Slc31a2, Phlda1, Yrdc, Asap1, Twistnb, Soga1, Trmt10c, Sept7, Ell2, Cebpz, Celf2, Tubb2b, Pnrc2, Etf1, Msn, Stx16, Cdv3, Sap30, Bet1l, Alcam, Scaf11, BC037034, Cd300lb, Klhl6, Cdk6, Ssb, Cct4, Mkrn1, Rxrb, Map7d1, Slc15a4, Rbm5, St3gal6, Nisch, Tnfrsf12a, Ehbp1l1, Phf20l1, Camk2n1, Nrip1, Eif3d, Mtmr14, Phf23, Krit1, Wac, Jmjd1c, Tnrc6a, Brd4, Pea15a, Rela, Xrn2, Hist1h4d, Phf3, Napa, Pla2g15, Nr3c1, Itpripl2, Elf1, Nes, Vhl, Pgs1, Il10rb, Trim35, Gars, Siah1a, Ncl, Zfp263, Mical1, Cdca4, Rnh1, Anxa3, Trim27, Tiprl, Mcrip1, Csnk1d, Tbc1d10b, Ccar1, Ak2, Trappc4, Prpf4b, Rnf19b, Bag5, Srsf3, Rsbn1l, Papola, Eif4ebp1, Frrs1, Sh2b1, Plxdc2, Pmaip1, Dnm2, Dnajc7, Cbx1, Mrps18b, Crlf3, Xylt1, Ogt, Atp6v1b2, Mknk1, Rbms1, Rnf215, Srp72, Pim1, Borcs6, Phc2, Cept1, Atxn2l, Gsap, Stk40, Dcun1d5, Golgb1, Id2, Taok3, Cxcl16, Slc25a33, Rexo1, Cap1, Sltm, Zc3h7a, Bod1l, Tmem115, Rab2a, Kcnk13, Snx1, Rhbdf2, Epb41l2, Gps2, Akirin1, Rhog, Kras, Txndc9, Sall3, Gna13, Brix1, Zmiz1, Pim3, Rasa4, Smarca5, 5430427O19Rik, Ddx39b, Dgkz, Cebpd, Dgkd, Tbc1d23, Plin3, Il6ra, Atf1, Nsd3, Calhm2, Tmem140, Arl6ip5, Arl1, Tnrc6b, Serp1, Cklf, Commd7, Maea, Wapl, Tnfaip1, Ghitm, Dctn4, Vdac3, Cops4, Fam105a, Cdk11b, Tsc22d4, Dctn2, Herpud1, Syf2, Adap2os, Cpeb2, Nptn, Smox, Akap13, Btbd9, Arid4b, Cd274, Rock1, Ranbp9, Fmnl3, Cxxc5, Prrc2c, Picalm, 2010111I01Rik, Dhx36, Smad7, Ppp1r12a, Cep170, Lpcat2, Ubap2l, Sbno2, Polr2c, Dennd4a, Pla2g4a, Mcfd2, Sh3bp2, Asns, Kmt5a, Rab35, Speg, Jmjd6, Ubap1, Cyth2, Asb6, Ctbp2, Skap2, Got1, Cers2, Ythdc1, Setd3, Utp11, Zfp787, Rbm26, Phax, 9930111J21Rik2, Psmc2, Cdkn2aip, Sesn2, Pdcd7, Ccdc12, Zfp644, Il21r, Tmed9, Ywhag, Arl14ep, Yae1d1, Vezf1, Eif4a2, Qk, Zfp639, Selenos, Ccdc174, Unc50, Hmgcl, Ncor1, Bptf, Selplg, Spsb2, Nop58, Zfp768, Rbpj, Rab6a, Aen, Luzp1, Tox4, G3bp1, B230219D22Rik, Vmp1, Zranb2, Med7, D1Ertd622e, Cdkn1b, Pttg1ip, Trim47, Eif3j1, Foxn3, Rab7b, Lrrc59, Gpr65, Ash1l, Clk3, Ubn2, Snrnp48, Nkiras2, Thrap3, Rpl7l1, Tfg, Kctd12, Nr1h2, Lyn, Eea1, Pkn1, Taf7, Cnot4, Ptp4a2, Sec62, Gm16286, Sphk2, Ccr5, Tmem86a, Abcd2, Nmd3, Snx24, Cyb5r1, Gspt1, Rnf6, Aim2, Dot1l, Cirbp, Mphosph10, Rab5a, Slc2a1, Fubp1, Iffo1, Rgs19, Dvl1, Tgfbr2, Sugt1, Mterf3, Tbc1d17, Rab40c, Rtcb, Akna, Arhgap27, Pag1, Arhgef1, Cdc42se1, Vim, Rap2a, Pak1ip1, Gnl3, Mogs, Snx17, Ubn1, Tbce, Plekhf2, Rab11fip5, Ccnt2, Dcakd, Nr2c2ap, Selenok, Polr2e, Orai2, Abraxas2, Ubald2, Klhl25, Adprhl2, Cd83, Slc29a3, Bcdin3d, Prpf40a, Hook3, Ypel3, Cdk12, Csf1r, Spop, Lsm14a, Bcl2l1, Ppp4r2, Arid1a, Kmt2a, Slc15a3, Tns3, Ube2k, Mia2, Dmtf1, Srsf9, Smc6, Anks3, BC028528, Mars, Efhd2, Ube2j2, Nup54, Gpcpd1, Mdm4, Mau2, Hacd2, Map3k14, Slc35e4, Chd1, Ier2, Rab10, Tanc2, Nsfl1c, Trabd, Nsmce3, Snw1, Myo1f, Nfe2l1, Cnppd1, Bnip2, Slco2b1, Gpr34, Rbm18, 0610030E20Rik, Litaf, Gtpbp6, Zfx, Siah2, Leng8, Tpst2, Rnf146, Ski, Ing1, Gga1, Dnajc21, Phospho2, N4bp2l2, G3bp2, Mgat2, Fiz1, Ncf2, Hvcn1, Napg, Ppp1r11, Ankrd12, Casp8, Atp2b1, BC005561, Zmym5, Nfkb1, Pkn2, Foxn2, Plaur, Blcap, Smndc1, Cars, Peak1, Nfkb2, Arhgef2, Dlst, Bcl2, Nop56, Plekhj1, Nufip2, Strap, Cdk18, Bach1, Ypel5, Eif4e2, Susd3, Cisd2, Pabpn1, Maml3, Nup62, Sipa1, Tnrc6c, Clec5a, Kansl1, Arfgap2, Pxdc1, Bmyc, Cul1, Cnot2, Chd2, Stk38, Plgrkt, Tmub2, Slu7, Safb2, Cd37, Mpp6, Ptpn2, Crebzf, Map2k1, Copb2, Creb3, Rsf1, Chst12, Rock2, Mtpn, Pcm1, Cnot6l, Clip2, Sertad1, Cdk8, Diaph1, Tor1aip1, Zfp131, Triap1, Naa50, Ppil4, Galnt7, Hist3h2a, Vps9d1, Ptges3, Bzw1, Rabep1, Pck2, Rabggtb, Riox1, Zfp330, Fbxw11, Git2, Elavl1, Cdkn2d, Srpr, Rbbp6, Naa35, Necap1, Pwwp2a, Itgb2, Smim7, Lhfpl2, Zfp292, Gpatch8, Fhod1, Gnaq, Mvp, Adam8, Abhd12, Nfkbib, Apobec3, Bcar3, Ppp2r2d, Smad4, Tcf4, Rhoc, Zc3h15, Ggps1, Cnih4, Rb1cc1, Rbm47, Ube2h, AI837181, Ccnt1, Ythdf2, Fam129a, U2surp, Rraga, Tbk1, Ccdc86, Hsp90aa1, Srp19, Ctcf, Rgl2, Mllt10, Map2k3, Myc, Lrrc8a, B3galt4, Nae1, Snx10, Rassf4, Clip1, Ube2g2, Leng1, Cln8, Frmd8, Pacsin2, Fes, Nsrp1, Fam45a, Zc3h13, Susd6, Kdm1a, Spryd3, Zranb1, Mitf, Fzr1, Chchd4, Ankrd17, Ddi2, Hk2, Lacc1, Fam129b, Ppan, Dlgap4, Zrsr2, Plekha2, Ssbp2, Trpc4ap, Zfp36, Atf6, Tmcc3, Zc3hav1, Atg16l1, Inpp5d, Vsir, Prmt2, Ccdc71l, Amz1, Atf5, Kdm2a, Scfd1, Batf3, Rest, Rasal3, Akap8l, Crk, Ccdc130, Ints12, Arid4a, Slc25a37, Zcchc7, Nfkbil1, Ptbp1, 2310011J03Rik, Swt1, Usp2, Ncln, Trp53, Tbc1d10a, Ube2z, Ubr2, Mettl1, Mcub, Ctsc, Naip2, Fbrsl1, Tial1, Yars, Lilr4b, Vrk2, Ccdc115, Rbm42, Ppp1r10, AI467606, Ric8a, Ophn1, Comt, Cacna1a, Mysm1, Zfp62, Pip5k1a, Fam46c, Srxn1, Mrps31, Ythdf1, Mob2, Map4, Ddx28, Nemf, Zfp36l1, Wdr43, Spty2d1, Ccdc9, Fbrs, Fxr2, Strn3, AC160336.1, Orc4, Elf2, Vgll4, Prpf39, Stxbp3, Tsc22d3, Acox3, Trim8, Pfkfb3, Taf1d, Fam133b, Gpank1, Srek1, Rsbn1, Rbm22, Esco1, Plekha1, Gtf2b, Npepps, Dapp1, Rnmt, Anxa7, Slc2a6, Pi4k2a, Banp, Nub1, Yipf3, Pwwp2b, Msl1, Slc23a2, Fbxl3, Tbc1d15, Paxbp1, Il4ra, 2410004B18Rik, Ubash3b, Abi1, Trafd1, Gm26532, Pisd, Mgat4a, Arih1, Rit1, Frmd4a, Pum1, Gmps, Zfp513, Zfand2a, Tardbp, Trmt2a, Klhl24, Hax1, Ccdc186, Clic4, Zfp397, Cdc5l, Card19, Zfp830, Rchy1, Rae1, Tatdn2, Vps37a, Trim28, Mark2, Osbpl8, Zfr, Luc7l, Trib1, Ubp1, Hist1h2bc, Celf1, Slc8b1, Ptk2b, Anapc16, Ormdl3, Lrif1, Ppcdc, Trim44, Znrf1, Aars, Zfp326, Uba5, Dnmt3a, Gch1, Ankrd10, Cdk13, Cab39, Rnf4, Zfp710, Trpv2, Ifi207, Pex13, Gna11, Smn1, Bfar, Pdlim4, Mak16, Usp16, Pald1, Gse1, Pmepa1, Uhrf2, Actr1b, Peli1, Slc12a9, Rab20, Poldip3, Ilkap, Igf1, Nbeal1, Phf14, Dnaja3, 1600012H06Rik, Snhg20, Ppm1a, Arhgap39, Kdelr2, Arhgap17, Safb, Atg12, Dyrk2, Nckap1l, Zmynd8, Zcchc11, Rnps1, Lactb2, Ptcd2, Ufl1, Nrros, Wtap, Sde2, Samd4b, Limd1, Keap1, Nelfb, Tmem168, Plk3, Supt20, Hmox1, Raf1, Alkbh1, Mef2d, Zfpl1, Sla, Tet3, Crebrf, Sar1a, Thumpd1, Crcp, Fli1, Tma16, Eed, Rcsd1, Dst, Bloc1s4, Dnajc1, Setd5, Csf3r, Zfp120, Atg101, Csnk1g3, Topors, Arid5b, Morf4l2, Taz, Myo1e, Senp2, Abcc5, Sp3, Cbll1, Tlnrd1, Adap1, Phgdh, Cldnd1, Lin37, Utp18, Camk1d, Ikzf1, Dapk3, Tnfrsf1b, Ascc3, Rnf166, Spryd7, Zfp800, Gpr107, Cabin1, Slain2, Tmem119, Tsen34, Lrrfip2, Dnajb9, S1pr1, Pnpla2, Ivns1abp, Fgd2, Nop14, Nlrp3, Rel, Ppfia4, Tmem230, Tifab, Stk17b, Ifnar1, Golm1, Arid3a, Yipf5, Dnase1l1, Vat1, Slfn2, Tank, Il17ra, Cyp4f18, Hck, Hivep3, Pbxip1 | Tyrobp, Rps29, Fcer1g, Rpl37a, Rplp1, Rps24, Rps20, Rps11, Rps10, Rpl35a, Rpl23, Rpl32, Rpl19, Rpl11, Rpl41, Rpl27a, Rpl30, Rpl28, Npc2, Rps23, Rpl21, Rps12, Rpl39, Rps27a, Rps4x, Tpt1, Rps21, Rps16, Fau, Rpl26, Rps7, Rps13, Rpl13, Rpl35, Rps19, Rpl34, Rpl18a, mt-Co1, Cox8a, Rps28, Rpl36, Rpl29, Rpl37, Rpl36a, Rps15a, Rps3a1, Rplp0, Cox4i1, Rplp2, Cox6c, Rpl38, Atp5e, Cox6b1, Rpl22, mt-Co2, Rps26, C1qb, B2m, Rps8, C1qc, mt-Co3, Selenop, Rps3, Rpl15, Itm2b, Atp5h, Chchd2, Uqcrh, mt-Atp6, Rpl31, Rps9, Rps5, Rpl9, Atp5l, Rpl6, Rpl24, Rps25, Pfdn5, Uqcr11, Rps14, Rpl7, mt-Nd2, Gm10076, mt-Nd1, Rps2, Gas6, Rpsa, Rps18, mt-Nd4, Cfl1, Hexa, Atp5j2, Lyz2, Cst3, Atp5g2, Ndufa13, Rpl14, Lamp1, Oaz1, mt-Cytb, Ubl5, Bst2, Arpc3, Rpl3, Ndufv3, Eef1b2, Dbi, Apoe, Elob, Tmem256, Timp2, 2010107E04Rik, Rps27, Rpl12, Serf2, C1qa, Ctsb, mt-Nd5, Rpl18, Atp5j, Rpl27, Clta, Ndufa1, Trf, Rpl10, Rpl7a, Rpl8, Eef1a1, Ndufc1, Aes, Gm1673, Uba52, Prdx5, Tmsb4x, Cox7a2, Ptpn18, Tma7, Rps17, Ndufa2, Rpl17, Ap2s1, Nedd8, Uqcr10, Tomm7, Snx3, Eif3f, Gabarap, Cox5b, Hint1, Lamtor2, Gapdh, Ndufb11, Syngr1, Rpl10a, Cox7b, Cenpx, Sptssa, mt-Nd4l, Ssr4, Cox5a, Ndufs5, Gm11808, 2410015M20Rik, Mrps21, Park7, Cyba, Scp2, Tmem160, Atp5k, Gpx4, Rpl23a, Cox6a1, Eif3h, Naca, Mif, Wfdc17, Atp6v1f, Cd63, Cox7c, Ndufb8, Ndufa7, Minos1, Atp5d, Eif3k, Bsg, Serpinb6a, Dad1, Gamt, Ndufb7, Aif1, Uqcc2, Lgals1, Rps15, Grn, Psmb8, Ndufb5, Ndufa5, Hcst, Trem2, Romo1, Atp5o.1, Myl6, Qdpr, Nenf, S100a1, Prps2, Ndufb10, mt-Atp8, Sec61g, Edf1, Cd68, Ndufa3, Ggh, Uqcrb, Ndufb9, Gm2000, Sem1, Cpq, Fcgr4, Smdt1, Rack1, Atox1, Mt1, Bag1, Rapsn, Cetn2, Pf4, Laptm5, Fcna, Atp5g1, Nme4, Gstm5, Spcs1, Aldh2, Gpx3, Cryba4, Ndufa4, Ppia, Pla2g16, Cd28, Arhgdib, Ifitm2, Akr1b3, Ctsd, Sdhaf4, Rpl27-ps3, Gm9493, Ddt, Mpst, Rps6, Sdf4, Cd93, Igfbp4, Rpl5, Blvra, Crybb1, Rtp4, Hadh, Fcgr2b, Atp6v0e, Chchd10, Dcxr, Ppib, Nme1, Wdr89, Bphl, Glmp, Gm10073, Taf10, 1110008P14Rik, Fam213a, Ybx1, Fabp5, Prdx4, Snrpd2, Snrnp25, Oas1a, Ptma, Ramp1, Pomp, Smim4, Man2b2, Stab1, Selenof, 1700003F12Rik, mt-Nd3, Borcs8, Iqgap2, Mrpl28, Rps12-ps3, Khk, Snrpg, Fis1, Smim1, Man2a1, Oasl2, Hspe1, Ndufs6, Rnf130, Prdx2, Ndufc2, Tmem9, Mndal, Rpl9-ps6, Selenbp1, Fkbp2, H2-D1, Hfe, Tecr, Cops9, Gltp, Phyh, Epsti1, Gm38843, Nbdy, Ap1b1, mt-Nd6, Glb1, Adgre1, Sord, Isg15, Hpse, Cmc2, Lst1, Rps27rt, Cenpa, Fam213b, Capzb, Vkorc1, Slfn5, Maf, Ifitm3, Cfp, Slc25a4, Hsbp1, Mgst1, Spp1, Tmem205, Slc25a5, Gpnmb, Fcgrt, Rida, H2-T22, Mcee, Cuta, Sdhc, Npl, Rpl36a-ps1, Cela1, Ltc4s, Ndufa11, Lrrc27, Sod1, Fcrls, Bola3, Ptgr1, Srp14, Nudt19, Pltp, Hip1, Eif3i, Adk, Hint2, Nop10, Itsn1, Mgst3, Eef1d, Ift22, Tmem258, Ccdc34, Atp5g3, Bex3, Zbp1, Atpif1, Srgn, Bscl2, Syce2, Akr7a5, Cd302, Fundc2, Cib1, Tmem106a, Ly6e, Calr, Cnpy2, Ethe1, Arhgap19, Psme1, Folr2, Ndufa12, Selenom, H2afz, Xaf1, Ddrgk1, Ccl9, Palld, Ifit3, Rab5c, Vav3, Stom, Lgals9, Calm3, Sp100, Arsb, Acat1, Usmg5, Rac2, Phpt1, Hacd4, Copz2, Ifi211, Tpp1, Abracl, Ctsf, Rgs10, Dgcr6, Tmem97, Ebpl, Etfb, Ube2l6, Gmpr, Mrps25, Rnaseh2c, Paox, Tmem9b, Tspan3, Mfsd1, Adam9, Ndufa6, Rpl10-ps3, Hsd17b12, Dpm3, Eif2ak2, Mrps24, Dtymk, Cryl1, Dynlt1f, Agpat3, 1810037I17Rik, Clec4a3, Rpl13a, Clcn5, Pfn1, Fabp3, Anapc13, Tnni2, Smagp, Hmbs, Ctla2a, Ldha, Rnf213, Slc9a9, Nt5dc2, Aoah, Sh3bgrl, Ccl7, Tmem106c, Uap1l1, March2, Nsa2, Lgals3bp, Igbp1, F630028O10Rik, Ifi203, Cnp, Glo1, Dera, Rnf141, Trappc6a, Rnasel, Ms4a7, Atp1b3, Pgk1, Rab34, Gm2a, Clec4a2, Slirp, Polr2f, Zbtb8os, Lrp6, Aph1c, Pcbd2, Pnkd, Wwp1, Parp14, Ifi27l2a, Fcgr3, Irf7, Ang, Ndufb2, Qpct, Rps27l, S100a13, Gmnn, Ctsl, Cnrip1, H2-K1, Trappc1, Clec4n, Acsl5, Selenoh, Snrpf, Bmp2, Hmgn1, Rrm1, Dpp7, Mrc1, Pet100, Cdk1, Gng12, Rpl22l1, Dctpp1, Stat1, Psmb9, Dab2, Smc2, Dnajc15, Clec12a, Gmfg, Fos, Apoc4, Cd38, Snx2, Cks1b, Mcm3, Incenp, Ifi27, Idh2, Cd52, Fn1, Tmem141, Glrx, Fam162a, Alox5ap, Smpdl3a, Ctss, Crip1, Tmsb10, Ccl6, Hmgn2, Ckb, Ldhb |
| 3 | Actg1, Tmsb4x, Ftl1, Anxa1, Plin2, Pfn1, Lgals1, Actb, Capg, Prdx1, Hmox1, Slc25a3, Lgals3, Calm1, Esd, Arpc3, Ccnd1, Adam8, Myl6, Ckb, Fth1, Vim, Cstb, Cfl1, Rhoc, S100a4, Serpinb6a, Ppia, Clic1, Aprt, Sh3bgrl3, C5ar1, Arpc2, Cdkn2a, Cd72, Rtn4, Gsn, Pcp4l1, S100a6, Tpt1, Akr1a1, Psmd8, Anxa2, Arpc1b, Actr3, Serf2, Rps27l, Ccl9, Mmp12, Rnh1, Eif5a, Myl12a, Rps27a, Cdc42, Ifitm3, Zranb3, Anxa5, Ifi27l2a, Spp1, Hspa8, Ndufa4, Cd36, Eif4a1, Gng5, Eef1a1, Prdx2, Chchd2, Emp3, Slc25a4, Cap1, Vdac2, Glrx, Lilr4b, Aes, Psmb6, Tagln2, Atp5l, Tmem171, Arpc5, Ywhah, Eid1, Cfp, Txndc17, Cndp2, Rac2, Hk3, Lpl, Rps18, Rps2, Mdh2, Plp2, Rps26, Sem1, Uqcrq, Eef2, Rtraf, Taldo1, Cox5a, Txn1, Clec12a, Pkm, Smdt1, Rpsa, Cyb5r3, Tubb4b, Tspo, Clec4d, Plau, Cystm1, Aldh2, Psmb5, Btf3, Atox1, Akr1b8, Ahsa1, Cox8a, Atp5g1, Lmna, Igf1, Mgst3, Cox7b, Atp5f1, Rpl32, Cox6b1, Rpl17, Ran, Psma2, Prdx5, Rpl27, Cox6c, Gapdh, Cdkn1a, Tubb5, Pmp22, Tbca, Cox7c, Cd52, Tma7, Fabp4, Cox7a2, Ftl1-ps1, Atpif1, Phlda3, Calm3, Ostf1, Cycs, Snx3, Basp1, Cmpk1, Atp6v0e, Slc48a1, Glipr1, Rps17, Tpm4, Atp6v1e1, Dok2, Ube2m, Ifitm2, Tkt, Vma21, Ndufc1, Folr2, Psmb2, Psma3, Cenpb, Srp14, Ndufa13, Capn2, Rap1b, Sh3bgrl, Usmg5, Litaf, Mrpl20, Capns1, Aldoa, Mrps14, Dbi, Bax, Fam96a, Vat1, Dynlrb1, Asns, Pgk1, Pgls, Cct2, Mdh1, 1110008P14Rik, Tmsb10, Atp5b, Ap1s1, Hist1h2bc, Nes, Hprt, Igfbp4, Smpdl3a, Uqcr10, Cct8, Nap1l1, Ndufb6, Stmn1, Pfdn1, Wdr1, Cct5, Tuba1c, 1810058I24Rik, Vapb, Cox6a1, Psma4, Mrpl33, Crip1, Unc119, Glrx5, Rexo2, Dctn3, Qpct, Pin1, Chmp5, Metrnl, Glrx3, Gipc1, Tbcb, Tubb2a, App, Pcna, Mrpl57, Cops9, Gyg, Ndufa9, Snu13, Csrp1, Ccl2, Lat2, Tom1, Rraga, Ctsb, Bak1, Pfkp, Msr1, Tmem106a, Gpnmb, Dera, Plaur, Nudcd2, Lyz2, Casp1, Fabp3, Gcsh, Lipa, Ccl12, Anp32b, Gamt, Ccl6, Por, Iqgap1, Flna, Ctsd | Malat1, Apoe, Rsrp1, Cx3cr1, Fcrls, Tmem176b, Marcks, Tmem176a, Csf1r, Zeb2, Srrm2, Rbm39, Mef2c, Son, Ly86, Qk, Zfp36l1, AC149090.1, Ctss, Stx16, Ankrd11, Ctsh, Junb, Nrip1, Rrbp1, Ly6e, Git2, Serinc3, Ash1l, Mertk, Abca1, Mbnl1, Neat1, Cst3, Peli1, Ogt, F11r, Kansl1, mt-Nd2, Zfp292, 2810474O19Rik, Ccnl1, Cep350, Ubn2, Pnisr, Chd7, Pmepa1, mt-Nd5, Fyb, Mtdh, Tcf4, Sash1, Gm26917, Ptbp3, Zmiz1, Ssh2, Rbbp6, Celf1, Arhgap45, Tmcc3, Akap9, Fcgr2b, Atp2b1, Ccnt2, Slc29a3, Mef2a, Pou2f2, Laptm5, C1qa, Kcnq1ot1, Zufsp, Itgb5, Kctd12, Gtpbp2, Gnaq, Ifngr1, Gpcpd1, Cxxc5, Olfml3, Fam46a, Zfhx3, Adcy7, Il10ra, Vgll4, Itch, Ttc14, Runx1, Gpr34, Arid5b, Clk1, Scamp2, Abcd2, Hook3, Man2b1, Cltc, Zfp644, Setd5, Chd4, mt-Nd1, Mtus1, Tgfbr1, P2ry12, Il6ra, Numa1, Cbl, Ypel3, Pde3b, Mycbp2, Ramp1, Jmjd1c, Mylip, Kmt2a, Kmt2e, Entpd1, Chd2, Cfh, Srsf5, Tab2, Rbm47, Klf6, Slc38a2, Arhgap17, Gm26522, Epb41l2, Cd33, Eif4ebp2, Foxn3, Macf1, Hk2, H2-DMa, Egln3, Smad7, Zfp36, Klhl24, Ier3, Zmynd8, Herc1, Csf2rb, Fnip1, Fam46c, Tigd2, Nktr, Nisch, Zcchc7, Tra2a, Pnn, Fos, Ep300, Tmem37, Ppp1r15a, Arhgap5, Sf3b1, Lrp6, Pkn2, Tmem87b, Rel, BC005561, Ankrd44, Eif4g3, Arid4b, Tnrc6a, Pnrc1, Krit1, Tnrc6c, Ktn1, Itpkb, Bmp2k, Ddx3x, Ophn1, Zbtb4, Frmd4a, Crlf3, Mafb, Tra2b, 1700017B05Rik, Fus, Rnf145, Thoc2, Ankrd17, AU020206, Tnrc6b, Arid1a, Rbm26, S1pr1, Phf14, Lpar6, Srek1, Rbm25, Susd3, Kdm2a, Sbno2, Vsir, Frmd4b, Tsc22d4, Ptgs1, Slco2b1, Tfe3, Scaf11, Phf20l1, Alkbh1, Larp4b, Nsd1, Map3k1, Arhgap31, Pum2, Ranbp2, Paxbp1, Snrnp70, Ski, Ifnar1, Mark3, Tmcc1, Srrm1, AC160336.1, Irf2bp2, Mysm1, Bhlhe41, Rnase4, Ikzf1, Gpr183, Stab1, Brd4, Cd86, 9930111J21Rik2, Aftph, Ccnt1, Nipbl, Prpf38b, Wac, Pum1, Luc7l2, Cnot4, Helz, March7, Fcgr3, Chd9, Lpcat2, Pnpla7, Selenop, Hivep3, Hlx, Ppfia4, Rb1cc1, N4bp1, P2ry13, Mindy2, Cdc40, Lcp2, P4ha1, Spag9, Ang, Crebbp, Mllt10, Synrg, Srgap2, Pxdc1, Golgb1, Dnajb14, Gm26532, Lpcat1, Map4k4, Skil, Asxl2, Ttc28, Tifab, mt-Co1, Rhoh, Dync1h1, Zbtb38, Ier2, Pik3r1, Lpin2, Dock4, Fam76b, Gpatch8, Ctc1, Spen, Pag1, Ttyh3, Bptf, Elf2, Picalm, Brd1, Cited2, Clip1, Herpud1, Ddx17, Bach1, Nr3c1, Slc16a6, Inpp5d, Ythdc1, Il4ra, Crybb1, Btg2, Gatad2b, Polr2a, Leng8, Nfam1, Slc3a2, Wdfy3, Tet3, Ncor1, Sel1l, mt-Nd4, Atad2b, Ankrd12, Soat1, Safb2, Atp8a1, Ppp1r12a, Clec2d, Rere, Chd8, Csf3r, Tnrc18, Diaph2, Zfp638, Rbm5, Rreb1, N4bp2l2, Hexb, Rsbn1l, Ppp1r10, Marf1, Tmem55b, Pcm1, Tob2, Cryba4, Chd6, Trpc4ap, Trf, Rasgrp3, Atxn2l, Fchsd2, Bod1l, Zfp36l2, Zfp652, Pbrm1, Mob1a, Rsf1, Fam193a, Ccdc88a, Snrnp48, Peak1, Slc7a8, Golga4, Xbp1, Arl4c, Snhg20, Mdm4, P2ry6, Pbxip1, Phip, Mga, Cflar, Cmtm6, Irf8, Csnk1e, Cdk13, Slc35f6, Stard9, Stat3, Rlf, Swt1, Prrc2c, Eml4, Setd2, Aff4, Baz1a, Gigyf1, Gm26740, Prrc2a, Cebpz, Senp2, Crebrf, Ubr2, Tiparp, Cdkn1b, Nsd3, Dock10, Ddx50, Maf, Smap2, Rnf216, Reep3, Lyst, Nfkb1, Atxn7l3b, Arl5c, Lhfpl2, Xiap, Fnbp4, Atxn2, Ankhd1, Baz2b, Cdk8, Zfp148, Rgl2, Trps1, Ulk2, Pias1, Zcchc6, Fcho2, Zscan26, Ubn1, Gapvd1, Golm1, Glul, Epb41l3, Zkscan3, Fkbp15, Taok1, Fgd4, Zbtb1, Clk4, Rnf111, Cnrip1, C1qb, Btg1, Cd34, Clcn3, Fam53b, Trip11, Prex1, Scd2, Kif13b, Rgs2, Fli1, Rexo1, Chd1, Ppp1r9a, Abca9, Btbd9, Uhrf2, Dennd4a, Ddx3y, Sbf2, Msl1, Lrp1, Tbc1d16, Topors, Gpr146, Ldhb, Zswim8, Wasl, Akap13, Man2a2, Cdk12, Stag1, 0610030E20Rik, Zfp397, Arid4a, Foxp1, Trpm7, Zfx, Slc9a9, Klf7, Btbd7, Crtc3, Klhl6, Brd8, Akap8l, Lyl1, Rgs10, Tns3, Phf3, Zcchc11, Cd164, Rcsd1, Plekhm2, Osm, Stk17b, Ppcdc, Tlr2, Aim2, Neurl3, Zmym5, Adap2, 4833420G17Rik, Fgfr1op, Ms4a6c, Snx29, Zbtb7a, Kat6a, Ivns1abp, Hvcn1, Ubash3b, Larp4, Rassf1, Dnajc13, Pabpn1, Baz2a, Rapgef6, Herc2, Tle4, Cebpb, Dmtf1, Safb, Zbtb20, Bbx, Rap1gds1, Kdm7a, Ddx5, Klf3, Tanc2, Ltc4s, Arglu1, Bclaf1, Gramd1a, Il16, Ccdc174, Adrb2, Dgkz, Mkln1, Birc6, Cabin1, Nfkbid, Zfp90, Luc7l3, Fbxl3, Dusp6, Cebpd, Sipa1l2, Dido1, Tnfrsf1b, Mia3, Nufip2, Tagap, Cspp1, Usp2, Taz, Zfp62, Gmfg, Taf1d, Arhgap19, Ep400, Clock, Chfr, Wsb1, Taf7, Il6st, Galnt1, Zranb1, Raph1, Rasal3, Rnf6, Paox, Myo5a, Slc25a36, Mxi1, Wwc2, Cebpg, Kdm5b, Gna11, Rsbn1, Upf3b, Dmxl1, Mef2d, Elf1, Il13ra1, Nfat5, Fam212a, Tspan14, Fam120a, Furin, Sox4, Smg6, Pan3, Abi3, Kmt2b, 4932438A13Rik, Hipk2, Usp34, Acin1, Mier1, Naa35, Zc3h13, Tec, Hnrnpul1, Angel2, Slc6a6, Smg1, Hps4, Mon2, Ccrl2, Rest, Ctsc, Rhoq, Zdhhc20, Snx30, Tjp1, Gon4l, Tgfbr2, Hacd4, Cd37, Vps13c, Zfp263, Hnrnpa2b1, AI314180, Bicd2, Thap3, Cmklr1, Gramd1b, Mafg, Npepps, Srsf11, Tpr, Hipk1, Dyrk2, Npc1, Elk3, Arid3a, Fam105a, Trappc10, F630028O10Rik, Stk38, Nfkbia, Gnl3, Rassf4, Dcaf8, Zfp704, Stk4, Dicer1, Ccdc88b, Rab32, Arhgap12, Tcof1, Apobec3, Enc1, St6gal1, Cep170, Kif21b, Snx18, Plxdc2, Nbeal1, Hps3, Ggnbp2, Efcab14, Pik3ca, Spty2d1, Fgd2, Znrf2, Ubap2l, Man1a2, Trrap, Pfkfb3, Abcc3, Myo9b, Fam133b, Cysltr1, Ssbp2, Slc38a1, Wwp2, Irf2, Brd3, Sipa1, Zfr, Washc4, Bdp1, Med15, Fnip2, Acox3, Pcnt, Etnk1, Cacna1a, Arhgap39, Brd2, Tbc1d17, Tia1, Zdhhc14, Wasf2, Ice1, Dnajc5, Ppil4, Trib1, Rnf2, Kdm5a, Pwwp2b, Gcnt1, St8sia4, Poldip3, Nfil3, Pwwp2a, Txnrd2, Ehmt1, Zrsr2, Zfp646, Ppm1h, Dst, Stard8, Tlr7, Cd82, Zfp445, Sfswap, Zc3h7a, Apc, Tnks2, Specc1, Itga6, Abr, Zmynd11, Hnrnph1, Ptprc, Xpr1, Lims1, Selenok, Prkcb, Adam17, Larp1, Ncor2, Tbc1d23, Med13, Adgb, Gns, Trim26, Cic, Fndc3b, Tbc1d12, Smc3, Tsc22d2, Tgif1, Mau2, Slc12a6, Kctd2, Rapsn, Rap2a, Cnot6l, Itpr2, Plekha1, Pcf11, Twistnb, Kmt2c, Sh3kbp1, Prpf4b, Osbpl11, Pds5a, Pom121, Prpf8, Vps18, B4galt1, Tmem243, Dctn4, Slc7a7, Rab14, Creb1, Fam208a, Mob3c, Arsb, Dusp1, Ldb1, Dusp7, Tmem86a, Bcl2l11, Kdm3a, Uvrag, Tmem57, Copa, Hist1h1e, Atrx, Dtx4, Secisbp2l, Atraid, Trmt1l, Abl1, Taf3, Ino80d, Zfp950, Ptprj, Rab11fip5, B230219D22Rik, Usp19, Cst7, Cd300a, Pik3cg, Smarca2, Med13l, Mfap3, Sec61a1, Bfar, Aup1, Sde2, Senp6, Acp2, Cep192, Tmem119, Snrk, Cd9, Rab20, BC037034, Mgat4a, Phf21a, Far1, Socs6, Tmem168, Csad, Comt, Traf3ip3, Ptpre, Plxnb2, Rbm6, Cd84, Smarca5, Lnpep, Synj1, Gpbp1, Nfkbiz, Ip6k1, Camk2n1, Rbm33, Nek7, Sh3bp2, Tmem268, Ubr4, Rassf2, Cntrl, Atf7ip, Ufl1, Atp1b3, Tcerg1, Nemf, BC017643, Wls, Brd9, Cwf19l2, Tmem50b, Gcn1l1, Atp2a2, Ctcf, Oxct1, Sowahc, Ankle2, Cnot3, Casp8ap2, Zfp830, Ylpm1, Pura, Ogdh, Epc1, Notch2, Iws1, Nfx1, Adam10, Eloa, Zfp326, Slc2a1, Zfp68, Arhgef6, Ap3d1, Camk1d, Usp12, 9930021J03Rik, Dnajc21, Vegfa, Rabgap1, Zfp512, Tgs1, Avl9, Phf20, Kin, Lcorl, Tnf, Sec62, Mpeg1, Vcpip1, 4931406P16Rik, Kif1b, Heca, Pip4k2a, Prpf39, Mroh1, Rbm28, Tgfbi, Gcc2, Csnk1a1, Man1a, Ddx6, Fbxw4, Retreg2, Irf2bpl, Zzef1, Lacc1, Slc38a9, Fam91a1, 0610040J01Rik, Pik3cd, Ago2, Clcn4, Slc4a7, Sesn1, Golga1, Ctdspl2, Rbm4b, Atxn1, Eif4g1, Plekhf2, Axl, Nudcd3, Map3k2, Pdcd7, U2surp, Sema4c, Tug1, Atp7a, Cnppd1, St6galnac4, Zfp800, mt-Co3, Slc12a9, Kdm3b, Cpne3, Strn3, Tnfaip2, Il17ra, Lsm10, Mtss1, Ccdc186, Cdc37l1, Nfrkb, Desi2, Tmem104, Smad4, Raf1, Flt1, Fmnl3, Trim44, Cpd, mt-Cytb, Dclre1c, Stag2, March1, Ing2, Smo, Vps9d1, Flcn, Dtx3, Mfsd5, Rtf1, Srpk2, Bhlhe40, Mark2, Phf12, Zfp710, Bambi, Klf13, Cirbp, Rad50, Gm38843, Cnot1, AW112010, Stxbp5, Ccnl2, Rcor1, Nfxl1, Sgpp1, Tbc1d14, Arrb2, Ttc3, Susd6, Ndel1, Ptpn1, Adap2os, Rock2, Znfx1, Spire1, Mitf, Scaf1, Plekhm3, Camsap1, Itgav, Samsn1, Crebzf, Prcp, Col4a3bp, Tmem131, Ints6l, Tacc1, Tcf12, Mphosph8, Taf15, Rps6ka1, Mdfic, Lsm14a, Rnf213, Rlim, Cyp4f18, Snhg12, Gse1, Nfic, Amfr, Mnt, Odf2, Icam1, mt-Nd3, Ago3, Tm9sf2, Gpx3, Bmpr2, Supt20, Scoc, Scarb2, Med1, Zmym4, Dhx30, Acap2, Phrf1, Eef2k, Ppp4r3b, Pkig, Ncstn, Naa15, Cd28, Arhgap4, Ilf3, Ubp1, Zfp622, Lilrb4a, Vps50, Dse, Mcur1, Gna15, Tex261, Etv3, Elmo1, Fam172a, Ntpcr, Ddx42, Cmtm7, Pald1, Jmjd6, Purb, Ddi2, Nupr1, Lrrc8a, Cep164, Erbin, Rnf150, Mknk1, Ccng2, Nrp1, Bsg, Zfp318, Mgat1, Rabep1, Sirt7, Man2a1, Nvl, Add1, Sppl3, Gngt2, Cd81, Usp25, Ate1, Trim8, Cttnbp2nl, Med7, Aoah, P2rx7, Nfix, Lrif1, Hnrnpl, Tmem106b, Esf1, Pkn1, Rtn4rl1, Zfp639, H1f0, Slc25a28, Bcl6, Rnf166, Alkbh5, Crk, Evi5, Nab2, Sbno1, Relb, Stau1, Usp7, Iars2, Naga, Sash3, Efr3a, Tgfbrap1, Ralgps2, Ero1l, Cldnd1, Hcst, Sgk1, Atp2c1, Stk24, Hif1a, Zfc3h1, Usf2, Psen1, Sp3, Snx13, Nck1, Smg9, Tbc1d4, 1700003F12Rik, Mfng, Tmx4, Fam168b, Pdpk1, Cd83, 2510009E07Rik, Bmyc, Huwe1, Zfp703, Usp16, Ddhd2, Dpy19l4, Clec4n, Ppp6r1, Rsrc2, Wipf1, Tm9sf4, Slc43a2, Aggf1, Reps2, Zfp106, Phf23, Bag5, Fndc3a, Itsn2, Slc29a1, Pnrc2, Rnf146, Pikfyve, Ikbkb, Ubl3, Pdk1, Top2b, Irf1, Prpf40a, Nsmce3, Upf1, Wdr43, Gtf2i, Tln2, Lman2l, Tpp1, Ccr1, Abhd6, Kdm2b, Mcmbp, Eif5b, Pdgfa, Itga4, Abcg1, Sf3b3, Kdm1a, Pcif1, Cmip, Magt1, Sema4d, Dnajc1, Usf1, Trip12, Tmf1, Fam102b, Baiap2, Dag1, Tnfaip8l2, Retreg1, Nlrp3, Lsg1, Trim12a, Yrdc, Mcrip1, Adipor2, Tox4, Pfkl, Coq10b, Ank, Sec14l1, Fubp1, Ccar1, Luc7l, Pon3, Rsrc1, Abi1, Bnip3, Sf1, Txlna, Slc15a4, Clec5a, Arhgef1, Gmip, Ncoa3, Fam76a, Gtf2h2, Igf2bp3, Colgalt1, Gna13, Ogfrl1, Faf2, Edem2, Akr1b10, Tbc1d20, Cd2ap, Zranb2, Arhgef2, Ythdf2, Kcnk13, Arhgap15, Hpse, Gpr84, Slc30a9, Camk2d, Cdc5l, Ppig, Egln1, P4hb, Saysd1, Pcyox1, Pygl, Dhx36, Gpr65, Hhex, Slk, Apbb2, Zfp91, Eea1, Lemd2, Sltm, Trim30d, Baz1b, Zfand5, Nav1, Tmem140, Tpst2, Ccni, Engase, Trim35, Tifa, Washc2, Lmo4, Fn1, Srsf2, Ppp3ca, Wapl, Tardbp, Eif5, Sntb2, Apoc4, Tpp2, Serpine2, Rock1, Ptafr, Itgam, H2-Q4, Atf1, Selenbp1, Fam114a2, Morc3, Pitpnc1, mt-Nd6, Prkcd, Aig1, Bbc3, Rasa4, Ms4a6b, Akirin2, Luzp1, Nfe2l2, Ifngr2, Mbnl2, Ier5, Slc16a3, Gm1673, Smox, Tcirg1, Bmp2, Mxd4, Rin2, Celf2, Mcl1, Cebpa, Apoc1, Hnrnpdl, Prps2, Rtcb, Ankrd37, Sfpq, Itsn1, Il21r, Ncoa4, Rnasel, Parp14, Gm2a, Cebpzos, Tecpr1, Srgn, Nrbf2, Hpgd, Srsf10, Apobec1, Wwp1, Ifrd1, Rgs1, Lgals3bp, Cybb, Scn1b, Rhob, Jun, Pid1, Selplg, Klf2, Ccl3, Slc11a1, Cd38, Id1, H3f3b, Id2, Ccr5, Pdgfb, Atf4, Mrc1 |
| 4 | Cd36, Adam8, Pcdh7, Clec4b1, Tmem171, Dpep2, Clec12a, Anxa1, S100a4, Ccl9, Akr1b8, Stap1, Abcb4, Dok2, Clec4d, Pcp4l1, Rtn4, Cdk14, Ifitm6, Cd72, C5ar1, Lpl, Igf2bp2, Iqgap1, S100a6, Dmpk, Nrcam, Pparg, Zranb3, Ahnak, Gsn, Capn2, Pqlc3, Ifitm3, B4galt6, Myo1e, Pid1, Cfp, Kitl, Plk2, Capg, Shtn1, Slc25a24, Ifitm2, Esd, Actr3, Lipa, 2010111I01Rik, Glipr1, Plin2, Flna, Txnip, App, Por, Plp2, Ifi27l2a, Eid1, Lilr4b, Clic4, Emp3, Siglec1, Igf1, Pls3, S100a10, Glrx, Oasl2, Nes, Ell2, Cd300lb, Cdk18, Metrnl, Samd9l, Cd300ld, Asph, Ckb, Cd44, Mcub, Irf7, Cd93, Fabp4, Glg1, Fxyd5, Gm4951, Tpm1, Pirb, Lgals3, Cybb, Msr1, Actg1, Col14a1, Alcam, Vav3, Tspan13, Phlda3, Pfn1, Nostrin, Myof, Lgals1, Cpne8, Rab7b, Serpinb6a, Hip1, Slfn2, Slc27a1, Slc35e4, Tspo, Fam241a, Tom1, Cnn2, Piezo1, Tcf7l2, Fnbp1l, mt-Atp6, Neat1, Nanos1, Lmna, Rhoc, Calm1, Ccnd1, Cystm1, Pam, Arhgap10, Ndufa4, Myl12a, mt-Co2, Cdkn2a, Cdkn1a, Mx1, Hmga1, Anxa5, Asns, Fam129b, Aprt, Rtp4, Myl6, Actn1, Lrrc25, Actb, Prdx1, Plekho2, Xylt2, Psen2, Cd5l, Ube2l6, Fcgr4, Apobr, Il3ra, Vma21, Cd52, Sh3bgrl, Dapk1, BC005537, Rnf128, Rnh1, Hmox1, Alox5ap, Mndal, Tmem120a, Tagln2, Ifi211, Rps27l, Cadm1, 1110008P14Rik, Psd3, Lyz2, Prdx2, Cdk6, mt-Co1, Phka2, Pfkp, Fth1, Trim25, Tbrg1, Isg15, R3hdm1, Xaf1, Hpcal1, Tes, Qpct, Ncoa7, Adssl1, Serpinb6b, Diaph1, Slc16a10, Smpdl3a, Evl, Plau, Slc37a2, mt-Co3, Stat2, Nav2, Ecm1, Sp100, Chd3, Malat1, Fam129a, Dab2, Ifit1, mt-Cytb, Unc119, Folr2, Mapre2, Fbxw17, Srxn1, Cyb5r3, Ifi209, Neurl3, Taldo1, Nav1, Cstb, Plaur, Bax, Ldlrap1, Sms, Basp1, Phf11d, Ets2, Mt2, H2-D1, Prr13, Map2k3, Oas1a, Ifi207, Samhd1, Adgre1, Lcp1, Stom, Tln1, Bmp2, Phf11b, Rsad2, Slfn5, Arhgef7, Abhd5, Rasa1, Ifit3, Slc48a1, Tspan17, Ptk2b, Ywhag, Slamf9, Mmp12, Pcna, Tbc1d1, Vim, Ifit2, F630028O10Rik, Txn1, Arhgap18, Tubb6, Nap1l1, Napg, Ehd4, Plec, Spag9, Cacna1a, Apobec1, Casp1, Tmem106a, Anxa2, Carhsp1, Tmem192, Ybx3, Anxa7, Slc40a1, Gcsh, Lfng, Itgb1, Nrp2, Dera, Vipas39, Cat, Itgb2, Parp14, Crip1, Hk3, Acvrl1, Nceh1, Spp1, Rnf213, St3gal5, Epb41, Ypel5, Card19, Fcgr1, Zyx, Mgst3, Sdc3, Mcm6, Ifi203, Peak1, Dusp1, Tpm4, Wdr1, Stat1, Fgd4, Id1, Clec2d, H2-T22, Fam20c, Tor3a, Gclm, Ccl6, Taok3, Cycs, Azin1, Igfbp4, Gas7, Ifi35, Hpgd, Gatm, Mdm2, Osbpl8, Csf2ra, Ifi204, Rgs1, Lat2, Hgsnat, Palld, Thap7, Cxcl14, Clec4a3, H2-T23, Clec4a2, Sdc4, Klf2, Lst1 | Fcrls, Ltc4s, Fcgr2b, Hexb, Gpr34, Tmem176a, Olfml3, Ang, Ctsz, Pmepa1, Rnase4, Bhlhe41, Ly6e, Ier3, Ldhb, Tmem176b, Bnip3, Mif, Scoc, Rgs10, Syngr1, Pfkl, C1qa, Cx3cr1, Apoc4, Ctsh, Apoc1, Ctss, Qk, Apoe, Pxdc1, Arsb, Kctd12, Epb41l2, Egln3, Ophn1, P2ry12, Marcks, Echs1, Npl, Ssh2, Ramp1, Gng12, Ptms, Lpcat2, Rps12, Ccl3, Abi3, Lgmn, Smap2, Gm1673, Cd81, Serinc3, Hk2, Scamp2, Zfhx3, Vegfb, Cxxc5, Mpc1, Crybb1, F11r, Bin1, Cd33, Tmem37, Ctsa, Egln1, Bsg, Mylip, Bmp2k, Ctsd, Cd9, Fcgr3, Csf1r, Itgb5, Cryba4, Cebpzos, Dtnbp1, St6galnac4, P2ry13, Ctnnb1, Eno1, Anxa3, Pgam1, Fam46c, Selenop, Tpi1, Oxct1, Gmfg, P4ha1, Glul, Ifi27, Tmem86a, Cst3, Atp6v1g1, Tlr2, Mtus1, Rab14, Pkig, Rps29, Ptgs1, Ypel3, Hebp1, Ntpcr, Serpine2, Gngt2, Rtcb, Ly86, C1qc, Tcn2, Mef2c, Gna12, Entpd1, Map4k4, Arl4c, Timp2, Rapsn, Comt, Mindy2, Lyl1, Zfp36l1, Scaf11, Lap3, Laptm5, Necap2, Hpse, Cd86, Slc16a3, Abhd12, Tuba1b, Atp1b3, Fam162a, Arl6ip1, Gde1, Gm2a, H2-DMa, Tmem119, Tagap, Man2b1, Cmtm6, Cd37, Cst7, Bmyc, Prps2, Pnrc1, Ivns1abp, Tgfbr1, Nr3c1, Atraid, Golm1, Cmtm7, Gns, Klhl6, Hmgb1, Tjp1, Scn1b, Mtdh, Dnajb14, Naglu, Csnk1e, Slc9a9, Cltc, Gpx3, Pde3b, Tpst2, Scd2, Slc3a2, Lmo2, Sirt2, Btg2, 1700003F12Rik, Tbc1d16, Gas6, Abcd2, Slc29a3, Paox, Fbxw4, Cd34, Reep3, Sptssa, Ccrl2, Vsir, Hvcn1, Lrp6, Epb41l3, Rnaset2a, Mcrip1, Arl5c, Tifab, Mertk, Arhgap5, Plek, Sgk1, Man1c1, Ctc1, Pld3, Dpp7, Fus, Ldha, Usp2, Skil, Slc16a6, Pla2g15, Rhoh, Man1a2, Rasal3, Arhgap45, Gramd1a, Chd7, Ulk2, Tgfbr2, Il4ra, Pbxip1, Pag1, Tspan14, Cebpz, Hnrnpul1, Ccl7, Gm26522, H2afz, Fchsd2, Tnf, Herpud1, Gna15, Maf, Gpr183, Chchd10, Inpp5d, Rhob, Ccr1, Gpr84, Ski, Samsn1, Fam212a, Peli1, AU020206, Slco2b1, Slc2a1, Ppfia4, Ppp1r15a, Mrc1, Cebpa, Cebpg, Junb, Fgfr1op, Cebpd, Cnrip1, Irf8, Hmgb2, Selplg, Hmgn2, Cd28, Smad7, H2afv, Fabp5 |
| 5 | Fau, Rplp1, Rps10, Rpl21, Rplp0, Rps26, Cst3, Rps12, Rpl10, Rpl7, Rpl18a, Rpl26, Rpl30, Rps9, Rpl19, Rpl23, Rpl13, Rps23, Tyrobp, Rps3a1, Rpl11, Rps24, Rps14, Rpl27a, Rps16, Rps13, Rpl9, Rps4x, Rpl35a, Rps11, Tmsb4x, Rplp2, Rps7, Rps5, Rps3, Rack1, Rpl8, Ctss, Rpl29, Rps15a, Rpl10a, Eef1a1, Rpl18, Rpl3, Rpl6, Rps18, Rps19, Rpl22, Gnas, Rpl32, Rpl35, Rpl15, Rps20, Rps29, Rps27a, Rpl34, Rps27, Eef1b2, Rpl17, Rpl36, Rpl28, Npc2, Rpsa, Rps21, Rpl7a, Rpl14, Pfdn5, Eef2, Uqcrh, Rpl39, Rpl37a, Rpl37, Rpl24, Rpl41, Rps25, Rpl12, Ssr4, Serf2, Eif3f, Cd52, Mpc1, Rpl36a, Tmem176a, Cd81, Hexb, Rps28, Rpl27, Tmem176b, Ldhb, Rps8, Rpl38, Sparc, Nsa2, Cox7a2l, Itm2b, Rps2, Olfml3, Sh3bgrl3, Rpl4, Rps6, Cst7, Naca, Gngt2, Ly86, Atox1, Rgs10, Gm10076, Ctsh, Eif3h, Uba52, Laptm5, Gm2a, Creg1, Eif3k, Ctsl, Pabpc1, Apoc1, Cd74, C1qa, Serpine2, Brk1, Itm2c, Rnaset2a, Ctsd, Cd34, Gm11808, Mrpl52, Syngr1, Rtcb, Aif1, Ppfia4, Vkorc1, H2-DMa, Fcrls, Selplg | Malat1, Zeb2, Rsrp1, Rbm39, Srrm2, AC149090.1, Neat1, Pnisr, Son, Rbm25, Cx3cr1, Mef2a, Stx16, Ankrd11, Ptbp3, Klf6, Ttc14, Nktr, Clk1, Zfp36l1, Pnn, Tra2a, Tcf4, Jmjd1c, Tra2b, Tmcc3, Kansl1, Ccnl1, Srrm1, Zmiz1, Ogt, R3hdm1, 9930111J21Rik2, Sash1, Dab2, Nisch, Gnaq, Junb, Fus, Ankrd12, Arhgap17, Ssh2, Zmynd8, Gtpbp2, Snrnp70, Hnrnpa2b1, Zfp292, Bod1l, Runx1, Gm26917, Camk2d, Frmd4b, Mcl1, Kcnq1ot1, Zfp36, Chd7, Lpar6, Zfp644, Gpcpd1, Nrip1, Bclaf1, Acin1, Ubn2, Nrros, Lipa, Rsbn1l, Zfhx3, Luc7l2, Git2, Ccnt2, Tpr, Rbm47, Luc7l3, Prrc2c, Arglu1, Pou2f2, Tnrc6a, Prpf38b, Trim35, Cd84, Mtss1, Eif3a, Rbms1, Pid1, Btg2, Rgs2, Apobec1, Zcchc7, Ash1l, Gm26522, Mafg, Kmt2e, Slc38a2, Eif5b, Rbbp6, Ubr2, Celf1, Rbm5, Cnot4, S1pr1, Krit1, Chd4, Id2, Mbnl1, Ube3a, Brd1, Pura, Arid4a, Nipbl, Slc15a3, AC160336.1, Ddx50, Arhgap25, Birc6, Zufsp, Peli1, March7, Arid5b, Atp6v0b, Sbno2, Srsf10, Abca1, Itpkb, Atp6v1a, Brd2, Tnrc6c, Zbtb7a, Ate1, Mycbp2, Ppp1r10, Ifnar1, Kdm2a, Wsb1, Mbnl2, Arhgap31, Ing4, Fyb, Cbl, Taok3, Stab1, Snhg20, Tnrc6b, Golgb1, Crlf3, Prpf40a, Ythdc1, Ccnl2, Taf1d, Wac, Tmcc1, Eif4g1, Srek1, Ier5, Kif13b, C3ar1, Irf2, Xrn2, Slc6a6, Pum1, Alkbh1, Bptf, Stk38, Peak1, Ier2, Rasgrp3, Trim8, Ankrd17, Uhrf2, Cited2, Pitpnc1, Cybb, Dnmt3a, Csf2rb, Lcp1, Herc1, Cebpb, Tab2, Srsf2, 2810474O19Rik, Ddx5, Rsrc2, Rhoh, Upf3b, Safb2, Arl4c, Fos, Phip, Stk17b, Arhgap30, Srpk2, Zrsr2, H3f3b, Ppp1r15a, Iqgap1, Tmem87b, Chd2, Tet3, Cdk11b, Srsf11, Smad7, Ndel1, Trip11, Tecpr1, Prpf4b, Phf3, Crebbp, Phf20l1, Ep300, Sirt7, Kmt2a, Btbd9, Ncor1, P2ry6, Rnf111, Susd6, Lrp1, Atxn2l, Ddx3y, BC005537, Cd180, Arhgef1, Itsn2, Fgd4, Rab20, Mertk, Uvrag, Nfe2l2, Rgs1, Polr2a, Hist1h1e, Itch, Irf2bp2, Usp15, Ddx42, Thoc2, Sel1l, Zfp36l2, Pkn2, Dock4, Rgl2, Cggbp1, Ptprc, Arhgap45, Rb1cc1, Adcy7, Elf1, Zcchc6, Snx24, Hnrnpdl, Rbm26, Sltm, Baiap2, Tfe3, Neurl3, Adap2, Zc3h7a, Nufip2, Esf1, Wwp1, Snrnp48, Srsf5, Paxbp1, Leng8, Tns3, Lrp6, Zbtb4, F630028O10Rik, Ap3d1, Asap1, Atxn7l3b, Tnfrsf1b, Rreb1, Dhx36, Gatad2b, Mkln1, Med15, Myo5a, Snx30, Gm26532, Eea1, Il10ra, Helz, Zc3h13, Frrs1, Gpr65, BC005561, Chfr, Hlx, Pabpn1, Baz1b, Brd4, Rrbp1, Tmem55b, Gapvd1, Arrb2, Ascc3, Gm26740, Atp6v0a1, Rel, Rexo1, Twistnb, Napg, Actl6a, Ktn1, Nsrp1, Rabep1, N4bp2l2, Srrt, Tnfaip8, Baz2b, Ddhd2, Sf1, Larp1, Ifi207, Larp4b, Slc35f6, Chd8, Jun, Macf1, Dnttip2, Sesn1, Msr1, Prrc2a, Dusp6, Pi4k2a, Kif5b, Arid4b, Srpk1, B4galt6, Zfp710, Sntb2, Arhgap10, Gramd1a, Zswim8, Irf2bpl, Klhl24, Mphosph10, Sf3b3, Mdfic, Rsrc1, Lyl1, Mtpn, Frmd4a, Metrnl, Ccar1, Nemf, Ccnt1, Dctn4, Arid1a, Gramd1b, Specc1, Gon4l, Sfpq, Srsf7, Slfn2, Rbm28, Gps2, Zfp397, Foxn3, Zfp638, Lpin2, Golga4, Naa35, Cspp1, Ranbp2, Stxbp3, Zfp704, Pbrm1, Gnl3, Mob4, Ggnbp2, Taz, Nrd1, Nsd3, Inpp5d, Tnrc18, Mia3, Nfkb1, Txnip, Atrx, Cdc40, Lcp2, Ifi204, Trim26, Rbpj, Rfc1, Zcchc11, Clk4, Cdc37l1, Thap3, Asxl2, Arhgap18, Mphosph8, Dock10, Ctcf, Tob2, Lilr4b, Sbf2, Kdm1a, Slc43a2, Nrp2, Hspa5, Cdc42se2, Ppp1r12a, Nbeal1, Smarcc1, Ufl1, Kdm7a, Mark3, Prkcd, Ccdc174, Luzp1, Nceh1, Slc7a8, Zfp207, Ubxn4, Mdm4, Soat1, Acbd3, Stard8, Mef2c, Sf3b1, Map3k1, Ankrd44, Pag1, Rmnd1, Tpp2, Mdm2, Ttc3, Spen, Glg1, Rsf1, Zfand5, Gpr183, Smad4, Nsf, Hsp90b1, Tcerg1, Snx1, Crtc3, Myo9b, Klf3, Zfp830, Pik3ca, Ehmt1, Xiap, 0610030E20Rik, Phf20, Rap2b, Dgkz, Snx29, Hectd1, Mysm1, Foxp1, Ehd4, Brd3, Atad2b, Mak16, Mau2, Serpinb6a, Fam120a, Fgfr1op, Setd2, Maf, Map4k4, Akap8l, Vav3, Aftph, Fnbp4, Cflar, Nfkbid, Fam111a, Wdfy3, Snx20, Gtf2b, Clic4, Arap1, Wasl, Ppp3ca, Kcnk13, Dmtf1, Slk, Ubap2l, Med4, Clec2d, Arhgef2, Nop14, Usp48, Chd9, Trps1, Atp2b1, Washc4, Luc7l, Ddx24, Setd5, Fam193a, Cln8, 1700017B05Rik, 4833420G17Rik, Ski, Angel2, Coq10b, Fkbp15, Phf14, Wdr26, Tcirg1, Hipk2, Tmem165, Cdk12, Sap30, Fam76b, Topors, Suz12, Itga6, Hnrnph1, Atf4, Naa15, Morc3, U2surp, Sf3a3, Tia1, 5430427O19Rik, Sox4, 2410004B18Rik, Hnrnpl, Nfkbia, Znrf1, Rasa1, Wdr12, Slc25a36, Chd6, Tigd2, Akap9, Tmem189, Cab39, Rnf6, Ubn1, Tnfaip2, Osm, Bcl2l1, Pafah1b1, Wbp11, Exosc3, Cep350, Rnf4, Swt1, Cdk6, Supt20, Abraxas2, Rasa4, Samhd1, Cenpb, Tm2d3, Trim30a, Igf2bp3, Gpatch8, Chd1, Vgll4, Zranb1, Zfp622, Snx2, Stag1, Ep400, Fam46a, Osbpl8, Chd3, Rab5b, Ubr4, Actn4, Ppp1r9a, Cd14, Ptpre, Sfxn1, Sp100, Smg6, Atg101, Cd47, Dcaf8, Bach1, Qk, Tnpo3, Senp6, Slc38a9, Nvl, Nrp1, Yrdc, Stard9, Ikbkb, Wipf1, Tmem140, Lmbrd1, Nsd1, Sh3gl1, Pias4, Gna11, Supt5, Cdkn1b, Klf13, Hivep3, Picalm, Larp4, Pikfyve, Rbm6, Tox4, Srgap2, Ifrd1, Lima1, Ccdc71l, Msl1, 2010111I01Rik, Virma, Tsc22d4, Usp34, Mark2, Ogdh, Heatr5a, Ddx46, Diaph1, Papola, Cnrip1, Spred1, Cdk8, Tm9sf4, Mgat5, Gcc2, Por, Kmt5a, Lnpep, Akap13, Vps18, Utp3, Lyst, Phf23, Arid3a, Clec12a, Tlk1, Numa1, Rab32, Pds5a, Zdhhc14, Arap2, Mier1, Fam133b, Vps50, Rere, Wwc2, Rapgef6, Iqgap2, Kif1b, Mllt10, Gigyf1, Ahcyl2, Nfe2l1, Cd93, Pdcd7, Kat6a, Top1, Tial1, Zfp148, Pcm1, Bbx, Eif4g3, Elmo1, Klf7, Snrnp40, Zkscan3, Dusp7, Tnfaip1, Faf2, Smc1a, Ing2, Trim12a, Fbxw17, Nfic, Mylip, C5ar1, Ppig, Bet1l, Plin2, Thrap3, Scaf1, Apc, Zc3h18, B4galt1, Trnt1, Strn3, Plk2, Dido1, Gpr146, Rest, Chordc1, Ddx3x, Tlnrd1, Puf60, Wdr43, Cd200r1, Lilrb4a, Eml4, Rnf214, Abcg1, Tiparp, Bfar, Rnasel, Clec4d, Spire1, Hip1, Slc29a3, Casp8ap2, Bcl2a1b, Tec, Oser1, Rnf216, Srsf1, Ubl3, Pias1, Trip12, Ppil2, Rcor1, Prkacb, Slc12a9, Ago3, Reep4, Swap70, Sh3bp1, B230219D22Rik, Nfx1, Acp2, Elf2, Arhgap19, Tbrg1, Wnk1, Osbpl9, Slc25a28, Zfp90, Dnajc13, Dlst, Rab14, Synj1, Ldlrap1, Cklf, Abi1, Smarca4, Trrap, Pcna, Nfat5, Trim44, Fnbp1, Mfsd12, Sdc4, Kif21b, Sec61a1, Samd4b, Desi2, Fam53b, Etnk1, Trib1, Ap2a2, Baz2a, Itpripl2, Fblim1, Rchy1, Ccdc86, Fam122a, Srsf3, Fcho2, Kif3a, Etv3, Slbp, A430005L14Rik, N4bp1, Ncoa3, Vcpip1, Cdc5l, Plekho2, Fubp1, Fchsd2, Plekhm2, Ttc4, Rnf149, Synrg, Ncoa7, Kin, 4932438A13Rik, Sirpa, Rlf, Tcf25, Tgif1, Parp14, Ankhd1, Ccdc59, Eif2ak2, Cnot1, Ophn1, 2510039O18Rik, Reps2, Nagk, Snx8, Nfam1, Pnpla7, Akr1b10, Rad50, Mroh1, Taok1, Ddi2, Gls, Mapk1, Smchd1, Phf21a, Dpm1, Cabin1, Rsbn1, Asph, Cep164, Pibf1, Spns1, Mrps5, Timm44, Rbm33, Nav1, Taf3, Pum2, Apbb2, Dst, Mndal, Usp19, Raph1, Spty2d1, Senp2, Gpr107, Bdp1, Mob3c, Pak1ip1, Tle4, Csnk1a1, Pwwp2b, Snrpa, Lactb2, Esco1, Raf1, Slc38a6, Fnip1, Cacna1a, P4hb, Dpp8, Sh3bp2, Chic2, Phax, Rufy3, Prex1, Gigyf2, Rnpep, Smc4, Nrbp1, Fnip2, Lrif1, Hmgcl, Wdr18, Ehmt2, Skil, Tmem259, Yy1, Myo1c, Sema4c, Yme1l1, Smarcc2, Rab3gap1, Tbc1d10b, Atp2a2, Iqsec1, Ccl3, Atxn2, Blvra, Med17, Zbtb20, Supt6, Srpr, Rab8b, Nmd3, Tbl1x, Slc16a6, Socs6, Kmt2b, Zfp106, Brd7, Fndc3b, Ppp4r3a, Sp110, Clock, Trappc10, Trim12c, Fbrs, Azi2, Creb1, Rab7b, Mapkap1, Gsk3b, Hnrnpd, Wwp2, Eloa, Dnajc1, Dmxl1, Fip1l1, Rbmxl1, 9930021J03Rik, Pik3cg, Zfx, Zfp91, Flcn, Keap1, Galc, Ttc28, Aff4, Nudcd3, Coro1c, Pik3r1, Pom121, Atp6v1h, Dgkd, Fbxo33, Zfp950, Trmt10c, Zmym5, Rnf213, Dse, Rtf1, Zfp703, Mgat1, Atp13a2, Plekhm3, Gtpbp4, Cebpd, Zfc3h1, Exosc7, Fam20c, Zdhhc20, Arhgef7, Taf15, Arhgap12, Ddx17, Zfr, Marf1, Zranb2, Huwe1, Tmem199, Map3k2, Nfrkb, Ddb1, Trpc4ap, Anxa4, Sh3bp5, Pnrc2, Abl1, Mplkip, 1110038F14Rik, Ccdc88a, Nr3c1, Trim25, Spsb2, Tardbp, Fer, Ankfy1, Ier3, Oasl2, Kmt2c, Rufy1, Zfp62, Ppp2r2a, Slc3a2, Ms4a7, Usp16, Ube2j2, Tsc22d2, Itga4, Bcl2l11, Unc119, Cdk14, Sptbn1, Mafb, Wdr91, Rsl1d1, Prpf39, Herc4, Smarce1, Osbpl11, Rassf2, Ing1, Fam76a, Xaf1, Cwf19l2, Rbm4b, Vmp1, Adss, Gna13, Hk3, Ranbp10, Ino80d, Tm9sf2, Cwc25, Cmip, Ppil4, Prkag1, Caml, Trpm7, Adipor2, Brox, Exoc4, N6amt1, Cd164, Eif4ebp2, Mrpl38, Pcf11, Acap2, Celf2, Med13l, Tbk1, Lrrc25, Lgals3bp, Ints6l, Cnppd1, Sipa1, Smc3, Cdk7, Ulk2, Zmynd11, Ice1, Stim1, Bcl6, Rraga, Rpp25l, Gtf2i, Upf2, Lacc1, Txnrd1, Phrf1, Csad, Noc2l, Tcf12, B4galt7, Knop1, Ppp6r3, Atg16l1, Rlim, Limd1, 2310035C23Rik, Gpnmb, Rnmt, Slu7, Lman1, Ptpra, Safb, Adgb, Trim27, Zfp646, Mbd1, Naa20, Zfp639, Dclre1c, Fyttd1, Fndc3a, Akirin2, Stard3, Slc9a3r1, Arrdc1, Hnrnpab, Ckap4, Mfsd14a, BC003965, Fbxl5, Rnf168, Tubb2a, Lrch3, Ikzf1, Baz1a, Npc1, Myo7a, Cd300lb, Ddx6, Tomm40, Hmgb2, 1700020I14Rik, Nacc2, Nop56, Zcchc8, Dera, Slc9a9, Cep192, Stxbp5, Rcc2, Abr, Vps4b, Smg9, Stat1, Irf3, Sde2, Med7, Arih1, Cadm1, Clcn3, Riok1, AI314180, Ring1, Cacul1, Ttc1, Tpst2, R3hdm2, Tbc1d20, Mob1a, Lrrc58, Man2a2, Nom1, Engase, Secisbp2, Twf1, Mef2d, Nae1, Zfp318, Rps6kb1, Adam17, Efcab14, Tbcc, Klf2, Zdhhc12, Stx4a, Ash2l, Zfp800, Fmn1, Smc6, Cyth4, Tgoln1, Ptafr, Dok2, Grn, Zfp652, Rhot1, Il21r, Atf6, St8sia4, Wdr20, Nrbf2, Slfn8, Gga1, Usp25, Dtx3, Poldip3, Hspa14, Epc1, Hps3, Tbc1d23, Gatad2a, Stat3, Tmem106a, Ccrl2, Rab24, Plod1, Abcc5, Mgat4a, Erg28, Adam8, Irak1, Cnot6l, Rars, Rnf126, Rilpl2, Cdk9, Slc17a5, P2rx7, Sf3a2, Taf7, Rtp4, Rabl6, Inppl1, Trim41, Dnm2, Agfg1, Nol11, Mat2a, Itsn1, Itgav, Cbx1, Ccr5, Nup62, Ifi203, Ddx41, Dnmt1, Hmg20b, Pitpnb, Khsrp, Hook3, Lhfpl2, Srsf6, Lfng, Ciz1, Ppm1h, Ppfia1, Ppp6r1, Gkap1, Bloc1s4, 4931406P16Rik, Prkcsh, Cntrl, Dusp1, Ak2, Zfp330, Zbtb38, Man1a, Ddx23, Zfp120, Csde1, Tagap, Hmgxb4, Zfp263, Irf8, Zscan26, Ythdf1, Btbd7, Dync1h1, Cfl2, Cep250, Arfgef1, Rab9, Mtus1, Ccdc186, Fbxo22, Cfp, Exoc3, Scfd1, Tmem70, Cir1, Dennd4a, Zbtb1, Atf3, Gdi1, Dnm1l, Dhx9, Uimc1, Sppl3, Ckap5, Slc30a5, Jmjd6, Plekhf2, Rabgap1, Eif4a3, Exosc8, Arhgap39, Dag1, Nfxl1, Nck1, Heatr6, Preb, Hps4, Zpr1, Tanc2, Gas2l3, Sfswap, Hnrnpm, Ankrd37, Pwp1, Lpxn, Id1, Lias, Mpp1, Plekho1, Xbp1, Gars, Ttyh3, Cebpg, Fam208a, Cttnbp2nl, Uhrf1bp1l, Evl, Syk, Utp11, Ubald1, Ywhag, Slc40a1, Mrps18b, Rapgef5, Stat6, Ifi211, Gdpd1, Ncstn, Ago2, Cast, Flna, Map7d1, Snx18, Tmx3, Brd8, Zfp24, Tmem206, Lrrcc1, Gak, Pmm2, Rock2, Sept7, Fbxw7, Necap1, Stx5a, Lcorl, Rps19bp1, Nufip1, Marcks, Impact, Dyrk2, Dhrs3, Cfap20, Psen2, Snx27, Icam1, Rab6a, Mga, Ubp1, Hpgds, AI987944, Palld, Fli1, Cd28, Ythdf2, Rassf1, Smn1, Zc3h15, Traf3ip3, Plekha2, Slc16a10, Bnip2, Saysd1, Trim47, Incenp, Prpf8, Med8, Rmi1, Ncor2, Cd44, Cirbp, Tbc1d17, Wls, Psmd12, Cd83, Cant1, Tmpo, Bag5, Ctsc, Slc12a6, Brix1, Copa, Slc30a9, Cyld, Smg1, Prpf19, Ip6k1, Tcof1, Snx9, Creld2, Enc1, Znrf2, Nsd2, Lactb, Spag9, Oxr1, Crebzf, Dcun1d5, Csnk1d, Mcm6, Il17ra, Tagln2, Scaf11, Mrc1, Rnf141, Arl8a, Ap3b1, Dnajc5, Clcn5, Smu1, Wdr61, Bambi, Man2a1, Tap1, Colgalt1, Kdm5b, Il6ra, Ubash3b, Ppp2r2d, Tbc1d1, Pdcd2, Epb41, Washc1, Sema4d, Parp2, Wtap, Hbp1, Kpna1, Odf2, Nab2, Dock8, Lsm8, Pmp22, Gar1, U2af2, Cnot8, Pnpla8, Aup1, Ubtf, Dars, Adap2os, Zfp68, Eef2kmt, Csnk2a1, Rap2a, Mrpl19, Slc38a1, Klhl9, Wbp4, Cd38, 2810004N23Rik, Mitf, Lmna, Ssbp1, Psmc1, Lasp1, Nfil3, Atxn1, Ifitm3, Dok1, Eed, Poldip2, Etv1, Xylt2, Plekha1, Ist1, Mrpl3, Lrrfip2, Ccl4, Trafd1, Ywhaz, Nkiras2, Ik, Filip1l, Dicer1, Ddx21, Prkcb, Ensa, Tango2, Nub1, Idh2, Tsen34, Lsg1, Atp6v1d, Mrpl32, Scyl1, Mogs, Cdk13, Camk1d, Dync1li1, Ddx27, Znfx1, Mapre2, Itgb1, Irak2, Adam9, Add1, Lgals1, Aplp2, Lemd2, Copb1, Ube2z, Ctnnbip1, Kctd12, Rab11fip5, Tspan14, Nlrp3, Lmo4, BC017643, Sbno1, Tnf, Eif1a, Hp1bp3, H2-M3, Nfya, Arhgef6, Lrrfip1, Eapp, Maf1, Agpat5, Cdv3, Polr2c, Fam212a, Pkn1, Ergic2, Tgfbr1, Susd3, Ppp4r3b, Myof, Csnk2a2, Mgst1, Mob2, Ilkap, Tom1, Wapl, Pbxip1, Gmip, Ppt2, Arhgap15, Arhgap4, Tmem11, Cep170, Txlna, Tpm1, Lars, Rnps1, Ralgps2, Psip1, Tln2, Vta1, Zc3hav1, Exoc5, Stag2, Crebrf, Timm17a, Tmbim1, Usp4, Sec14l1, 2610507B11Rik, Rock1, Ttc32, Zfp326, Ncl, Aagab, Mfhas1, Bcl10, 3830406C13Rik, Usp9x, Rdx, Ppcdc, Prpsap1, Herc2, Dnajc8, Fam114a2, Ms4a6b, Ehbp1l1, Ube2b, Lsm3, Fh1, Psmd7, Cars, Sat1, Diaph2, Pdk1, Med21, App, Arl8b, Slc15a4, BC003331, Slc2a1, Trmt6, Ewsr1, Il6st, Htatsf1, Ncf1, Srp72, Tmem86a, 2510009E07Rik, Marcksl1, Ccnd1, Cdc42ep3, Ptk2b, Rpn1, Pwwp2a, Irf7, Csf2rb2, Cyth2, Ripk1, Vegfa, Pcmtd1, BC037034, Ubc, Magt1, Tubb6, Tars, Secisbp2l, Acer3, Sowahc, Crk, Cenpa, Ifi35, Aph1c, Rnf2, Kdm2b, Aga, St3gal5, Myo1f, Pdhb, Ypel5, Slc31a2, Ms4a6c, Snw1, Pdia6, Aim2, Id3, Washc2, Btg1, Glyr1, Pla2g4a, H2-Q4, Ap1b1, Miip, Anp32b, Krcc1, Atad2, Sh3bgrl, Tubb4b, Usp7, Ddit3, Acbd5, Ncaph2, Epb41l3, Snrpd1, Ube2s, Mybbp1a, E2f1, Mat2b, Paox, Plaur, Phc2, Stip1, Pf4, Arf6, Rab5a, Tmem104, Topbp1, Wasf2, Nt5c, Psmc6, Dusp11, Nipa2, Il4ra, 2210016F16Rik, Supt16, Rin2, Fermt3, Calr, Nsmce3, Appl2, Nop58, Dnase1l1, Irf9, Uchl5, Dcakd, Ehd1, Hat1, Pgs1, Hnrnpul2, Nasp, Nckap1l, Gmnn, Rabggtb, Slc39a7, Dbf4, Eif5, Herpud1, Cltc, Psmd11, Stap1, Adrb2, Eif4a2, Atf1, Dtymk, Purb, Ahsa1, Pdgfb, Bmp2, Gla, Lamtor3, Tor1aip1, Usp1, Cdkn1a, Rcsd1, Map2k3, Isg15, Tcea1, Slfn5, Snx6, Tmx4, Rad21, Carhsp1, Acat1, Npepps, Nucks1, Dusp3, Bbc3, Lims1, Pfkfb3, Sgpl1, U2af1, Hist3h2a, Kras, Rassf3, Lrrc59, Smc2, Morf4l2, Eif2s1, Slc37a2, Parp1, Actg1, Tbxas1, Colec12, Bst2, 1810011H11Rik, Ell2, Rrm1, Plxnb2, Cd3eap, Eps15, Tgfbr2, Cdk1, Cdkn2d, Fcna, Samsn1, Fgd2, Ccr1, Sgk1, Sqstm1, Flt1, Tipin, H2afx, Fen1, Tuba1c, Ddx39, Gas7, Ccdc34, Sms, Sdc3, Cstb, Cks1b, Hmgn2, Kpna4, Ptpn1, Apobec3, Hk2, Mid1ip1, Tifa, Cela1, Cyb5r3, Sae1, Gsn, Hpse, Retreg1, Bex3, Ccl2, Stmn1, Lsm2, Dnajc9, H1f0, Fcgr2b, Rhob, Igfbp4, Rassf4, Dek, Hist1h1c, Clec4n, Ccl7, Mcm3, Slc16a3, Pdgfa, AU020206, Rapsn, Eprs, Csnk1e, Mthfd2, Folr2, Lgals3, Vat1, Cks2, Glipr1, Spp1, Tlr2, Rtn4, Ifitm2, Fabp3, Hspa9, Hmox1, Gclm |
| 6 | Ube2c, Birc5, Top2a, Mki67, Cenpf, Pclaf, Cdca8, Prc1, Tpx2, Hmmr, Cenpe, Nusap1, Ccnb2, Cdca3, Ccna2, Cdkn3, Tk1, Racgap1, Ccnb1, Tacc3, Cdc20, Spc24, Pbk, Kif20b, Knstrn, Plk1, Kif11, Kif23, Spc25, Knl1, Anln, Aurka, Ckap2l, Ndc80, Bub1b, Nuf2, Kif15, Kif22, Sgo2a, Esco2, Kif4, Kif2c, Cdca2, Aspm, Pimreg, Shcbp1, Kif20a, Ncapd2, Bub1, Dlgap5, Cep55, Cit, Diaph3, Lockd, Melk, Ncapg, Cenpm, Ccnf, Ckap2, Sgo1, Ect2, Kifc1, Neil3, Foxm1, Cenpp, Hmgb3, Nek2, Ska1, Trim59, Spag5, C330027C09Rik, Zwilch, Troap, Depdc1a, Ankle1, Prr11, Eme1, Mxd3, Sapcd2, Cdc25c, Ttk, Kif14, Ska3, Kif18b, Rrm2, Cenpw, Rad51ap1, Parpbp, Pif1, Cenpn, Mis18bp1, Oip5, Kif18a, Rad51, Mns1, Arhgef39, Mastl, Cenpq, Aurkb, Asf1b, Efcab11, Ccdc18, Fbxo5, Fam83d, Mad2l1, Cdc25b, Mtfr2, Cenpi, E2f8, Smc2, BC030867, Cks2, Cdca5, Lmnb1, Ncaph, Cks1b, Brip1, Poc1a, Spdl1, Cdkn2c, Lrr1, Trip13, Hist1h2ak, Cenph, Hist1h1a, Ska2, H2afx, Ezh2, Hist1h1b, Cenpk, Traip, Kntc1, Clspn, Gen1, Aunip, Stil, Plk4, Ube2t, Pkmyt1, Tyms, Ercc6l, Mcm10, Cenpu, Pmf1, Rrm1, Bora, Hist1h2ab, Tmpo, Smc4, Hist1h2bj, Cenpa, Cdk1, Brca1, Hmgb2, Stmn1, Incenp, Hist1h2ae, Rad54b, Espl1, Rad54l, Dbf4, Gpsm2, Rfc5, H2afv, Lbr, H2afz, Ube2s, Hmgn2, Ccdc34, Tedc1, Bard1, Tuba1b, Hmgb1, Slc43a3, Psrc1, Hirip3, Arhgap11a, Nucks1, Ndc1, Rangap1, Tubb5, Lsm2, Selenoh, Tubb4b, Chaf1a, Cenpl, Hist1h2ap, Hjurp, Haus5, Uhrf1, Hist2h2ac, Ran, G2e3, Hist1h1d, Nrm, Dsn1, Dnajc9, Iqgap3, Cdkn2d, Ccsap, Haspin, Exo1, Mybl1, Pole, Anp32b, Ptma, Ckap5, Sae1, Dynlt1f, Rad21, Cenpc1, Suv39h2, Rdm1, Tagln2, Sass6, Mis18a, Reep4, Blm, Anp32e, Usp1, Fignl1, Haus4, Lsm5, Lmnb2, Ncapg2, Jpt1, Rfc4, Rnf26, Gmnn, Tuba1c, Cep128, Dynlt1a, Hyls1, Cdc45, Nde1, Ppia, Rad18, Tcf19, Cbx5, Snrpd1, Hmgn5, Fen1, Gins2, Ulbp1, 4930579G24Rik, Kpna2, Hist1h3e, Ncapd3, Cmc2, Cenps, Cep89, Cntln, Arl6ip1, Raet1d, Nt5dc2, Hint1, Psmc3ip, Hist1h4d, Mcm5, Dek, Mcm8, Wee1, Cbx3, Brca2, Mcm7, Dnph1, Prim1, Suv39h1, Nup37, Pola1, Ddx39, Psip1, Ncaph2, Alyref, Hmgn1, Cdk5rap2, Vrk1, Miip, Rcc1, Txn1, Exosc8, Vars, Nudc, Anapc5, Ppih, Dtymk, Haus1, Spata24, Cep295, Gm42031, Smc1a, Prdx4, Csrp1, Rbbp8, Ddx11, Cep57l1, Topbp1, Lsm3, Rbm3, Mrpl51, Itgb3bp, Haus3, Rhno1, Lsm4, Snrpe, Hnrnpab, Ccdc77, Gins1, Rbl1, Ttf2, Chek2, Syce2, Hist1h4i, Mms22l, Brip1os, Calm2, Dhfr, Lig1, Mad1l1, Nsd2, Rpa3, Slfn9, Rpa2, Nup85, Snrpb, Ranbp1, Eif1ad, Bub3, Hdgf, Anapc11, Faap24, Tubb6, Atad2, Cenpt, Ssrp1, Nemp1, Nup43, Tbc1d31, Shmt2, Gm42047, Ilf2, Terf1, Nap1l1, Aaas, Banf1, Rbbp7, Pole3, Dut, Odf2, Myef2, Sumo2, Atad5, Mrnip, Rnaseh2c, H2-Q4, Uevld, Pcnt, Gcat, Gas2l3, Ubald2, Hist1h3d, Rbmx2, Nsmce1, Brd8, Srsf3, Cep192, Lnpk, Mndal, Prim2, Tmem97, Ybx1, Smchd1, Lin9, Nup35, Lin54, Hnrnpa3, Alg8, Cox7a2, Tubg1, Hat1, Rfwd3, Sf3b5, Apobec3, Hspa14, Hpf1, Cep70, Fzr1, Hist1h1e, Hnrnpa1, Srsf7, Pold1, Nasp, Ccne1, Erh, Timm50, Cenpx, Uchl5, Impa2, Ubl4a, Nsmce4a, Arhgap15, Pradc1, Mrpl18, Nmral1, Smc3, Taf5, Nav2, Carhsp1, Idh2, Tubd1, Dnmt1, Lsm8, Nudt21, Dnajc10, Psat1, H2-Q7, Uqcr10, Ifi203, Lbp, Haus6, Psmb8, Ift27, Xpo1, H1f0, Cep57, Gemin6, Cep112, Cox5b, Ctcf, Hnrnpa2b1, Thoc7, Cmss1, Rbmx, Ssna1, Tmem144, Rnaseh2a, Phgdh, Mapre1, Raly, Mcm4, Cryl1, Orc6, Snrnp25, Snx2, Syne2, Psmg2, Hnrnpu, Cdkn2aipnl, Psmb9, Tex30, Atp5o.1, Nudt5, Hsp90aa1, Tap1, Grk6, Nudt4, Smc6, Alg6, Sfpq, Ppil1, Iqgap2, Jpt2, Mrpl42, Tipin, Oscp1, Gle1, Lmf2, Ezr, Snrpf, Trim37, Calm3, 0610010K14Rik, H3f3b, Nedd1, Hist1h4h, Fam110a, Pop4, Elof1, Pcbd2, Snrnp40, Dazap1, Snrpg, Hp1bp3, Fam111a, Nop58, Cdk4, Rpa1, Srbd1, H3f3a, Mrpl28, Atp5j, Phf10, Ugdh, Nfatc2ip, Cdc123, Prelid1, Ndufab1, Rfc2, Skp2, Pbdc1, Eny2, Odf2l, Rnaseh2b, Nup205, Nup93, Psph, Snrpd2, Dpy30, Tmem138, Pds5b, Ndufa5, Pold2, Dck, Saal1, Egfl7, Ccdc61, Pih1d1, Ddx39b, Rdx, Mre11a, Igfbp4, Pck2, Adk, Sms, Med30, Larp7, Pfdn6, Hacd4, Pom121, Dctpp1, Pnp, Tfdp1, Ttc32, Bckdk, Mrpl33, Slc25a10, Rad50, 1810037I17Rik, Cntrl, G3bp1, Apip, Rpl22l1, Chaf1b, Ranbp2, Lcorl, Fkbp3, Ndufa4, Pa2g4, Pdzd11, Dnajc19, Nup160, Sephs1, Pdap1, Haus8, Zcwpw1, Srrt, Nfyb, Bcas2, Cby1, Phf6, Set, Nrf1, H2afy, Hells, Metrn, Atp5f1, Ssb, Nubp1, Bex3, Tagap, Cep83, Pspc1, Fmr1, Magoh, Trappc1, Clic1, Acat1, Wbp11, Anapc1, Sclt1, Cbfb, Ccdc88a, Ilf3, Ube2e3, Fam92a, Supt16, Nelfe, 1700025G04Rik, Snrpa, Atp5g3, Cmtm7, Nudt1, Mthfd2, Snrpc, Tpst1, Apmap, Sf3b2, Tubgcp2, Reps1, Rnf141, Mcm2, Mb21d1, Znhit3, Wdr76, Nol7, Atp5j2, Gins4, Sec14l1, Mrpl13, Dcps, Nqo2, Stip1, Fcna, Fbxl8, Eri1, Tma7, Ruvbl2, Fopnl, Asrgl1, Mtmr14, Atpif1, Lyar, Srsf1, Pbx3, Eftud2, Mcm3, Ndufb9, Mrfap1, Ywhaq, Babam1, Impdh2, Med9, Tcerg1, Ppp1cc, Nxt2, Psmb2, Tgfbi, Tmco1, Pde6d, Cep44, Rpp30, Pf4, Nxt1, Ndufb11, Ak6, Ptgr1, Acp1, B230118H07Rik, Cav2, Mum1, Cbx1, Tmem237, Nono, Grap, Nhp2, Psmc3, Fam96a, Mrpl49, Oat, Mycbp, Zwint, Stub1, Mcph1, Uqcrq, Actn4, Mrps14, Gmfg, Hnrnpr, Myg1, Rbbp4, Gpaa1, Nme1, Fmc1, Ccl7, Swi5, Sgf29, Pank2, Npl, Med4, Anapc7, Uba2, Suz12, Rps27l, Qpct, Psmc1, Rheb, Ubap2, Xrcc1, Cse1l, Etaa1, Parp2, Smdt1, Tssc4, F13a1, Crybg3, Tceal9, Sf3a3, Snrpa1, Pcna, Ywhae, Jdp2, Sf3a1, Smarca5, Uchl3, Id1, Kmt5a, Commd10, Thrap3, Phf5a, Akirin2, Cln6, Nup62, Cdc27, 2610524H06Rik, C330007P06Rik, Atp5b, Fundc2, Nsmce2, Tpr, Lsm6, Ruvbl1, Sf3b6, Cfp, Mars, Arpp19, Coq7, Mrc1, Ndufaf2, Ccr1, Zcrb1, 0610009O20Rik, Chchd6, Cnep1r1, Rfc3, Sept11, Sephs2, Prkag1, Ssr2, Tdrkh, Pcbp2, Cep290, Dzip3, Snrpd3, Pcyox1, Trappc13, Tmem256, Mcm6, Eefsec, Tsn, Cltb, Ndufb3, Gm8186, Serbp1, Usp39, Pkig, Hnrnpf, Rbm15, Carnmt1, Asxl1, Igf2bp3, Fh1, Taf6, Ppp1r35, Cwc15, Mettl9, Gtf2h5, Coa3, Stom, Uhrf2, Pla2g16, Asf1a, B9d2, Gnb2, Bcl7a, Med19, Prpf31, Trim28, Kpnb1, Mrps25, Polr2j, Leo1, Zcchc17, Mis12, Prpf38a, U2af1, Ap2s1, Pold3, Aoah, Ewsr1, Aars, Mrpl57, Sf3a2, Cfap20, Mmd, Nin, Yif1b, Gtf2a2, Dcp2, Cnot1, Fkbp2, Hcfc1, Odc1, Erg28, Etfb, Usp14, Ptpa, Cox20, Cnih4, Sun2, Anp32a, Ndufa11, Mtf2, Cdk19, Atg101, Phf11d, Sarnp, Lgals1, Hdgfl2, Psmb7, Cdk2ap1, Nfkbid, Palld, Lage3, Tmx2, Psmd9, Ccdc25, Gipc1, Commd1, Hdac1, Ndufa12, Msl3, Cfap36, Hdac3, Dnajc4, Med21, Polr2f, Lrrcc1, Maz, Srrm1, Cycs, Ndufc1, Cela1, Txndc12, Dnajc8, Nup50, Isca2, Ddx41, Cnot6, Psmd13, Med28, Hdac2, Tardbp, Rad23a, Prmt1, Psmc4, Oard1, Vps35, Prpf40a, Timm22, Cspp1, Brd9, Ppp2r2d, Clec12a, Mrps12, Utp3, Hikeshi, Smarcc1, Mprip, Fn1, Chchd1, Rnf168, Nudcd2, Psmd14, Ccnh, Srsf2, Mrpl55, Smarca4, Itsn1, Baz1b, Syncrip, Kif2a, Pin1, Prpf19, Mdh1, Hnrnpd, Tmsb10, Kdelr2, Paics, Cisd1, Zfp91, Metap2, Ogfrl1, Arhgap19, Rsrc1, Tmx1, Taf15, Ccl2, Cenpb, Nop10, Ppp1r7, Timm17b, Hmbs, Tnf, Usp25, Ndufv2, Trappc5, Eloc, Pim1, Numa1, Mrps18c, Eif1ax, Pnrc2, Mtch2, Vps36, Idh3a, H2-T22, Minos1, Cnih1, Csnk2b, Blvra, Thoc3, Tcof1, Tcp1, Hnrnpm, Ak2, Ndufv1, Hist1h1c, Rnf126, Rnps1, Ergic2, Vbp1, Srpk1, Cggbp1, Taf12, Gtf3a, Bcl7c, Atp5k, Bud23, Folr2, Upf3b, Hnrnpul2, Sptbn1, Cd38, Rif1, Actl6a, Slk, Bola1, Taf1, Ptges3, Anapc15, Siva1, Mpp6, Cenpv, A430005L14Rik, Dnajc15, Rnf4, Nupr1, 0610009B22Rik, Baz1a, Eif4e, Plod1, Ddah2, Polr2a, Pbrm1, Bud31, Lars, Brd3, Dera, Dok2, Mapkap1, Ifi27, Hnrnpl, Etfa, Rnf5, Casp8ap2, Paox, Ifi27l2a, Mrps26, Srsf10, Prrc2a, Nans, Osgep, Hnrnpul1, Pnkp, Phip, Mrps28, Bola3, Abracl, Ndufa3, Suds3, Slc38a2, Ndufs8, Ptbp1, Pcm1, Hnrnph1, Gspt1, Ppp5c, Tpm4, Ccar1, Maf, Ms4a6c, H2-M3, Tmem109, Phf11b, Stk38, Rel, BC005537, Galnt1, AC160336.1, Ppm1g, Ehmt2, Hpse, Tra2b, Eif4a3, Slc29a1, Rfc1, Kras, Pgp, Pttg1, Stag1, Atrx, Milr1, Scd2, Matr3, Dynll1, Slbp, Sap30, Parp1, Nop56, Gatm, Clec4d, F630028O10Rik, E2f1, Ccl12, Ramp1, Hgsnat | Itm2c, Rpl10, Cd63, Ctsz, Cd300c2, Fcgr3, Itm2b, Cd14, Ctss, Ucp2, Ypel3, Igf1, Cebpa, Fth1, Tyrobp, Creg1, Ctsl, Rabac1, Mpc1, Cd37, Btg1, Hexb, Rps27, Plxdc2, Eef1a1, Trem2, Vsir, Ctsh, Npc2, Cst3, Gpr34, Tnfaip8l2, Pnrc1, C3ar1, Ubc, Slc11a1, Psap, Efhd2, Jund, Ftl1, Anxa3, Fabp5, Mpeg1, Rhob, Rpl7, Cd53, Laptm5, Ctsa, Map1lc3a, Lpl, Junb, Ctsd, Tprgl, Sgk1, Mafb, Sirpa, Atox1, Pld3, Sdcbp, Plek, Asah1, Vat1, Cd84, Atp13a2, Abhd12, Cd72, Smap2, Gns, Bnip3l, Unc93b1, Cd52, Dhrs3, Serpine2, Slc6a6, Atf3, Cndp2, Grina, Bcl2a1b, Ptpre, Lamp2, Apbb1ip, Evi2a, Cd83, Orai1, Glul, Cd81, Ckb, Aprt, Cyth4, Ifngr1, Saraf, Tgfbr2, Tpd52, Selenop, Arl6ip5, Tmem176a, Btg2, Rftn1, Irf8, Bhlhe41, Sqstm1, Arhgap45, Hk2, Eif4a2, Ctsb, Aldoa, Rrbp1, Abca1, Ehd4, Cd9, P2rx4, Nceh1, Mylip, Tnfsf12, Slc15a3, Ldhb, Comt, Gngt2, Camk1, C5ar1, Capg, Ccr5, Cd300a, Gm2a, Pou2f2, Adap2, H2-T23, Timp2, Ptms, Akirin1, Pdgfb, Itgb2, Cstb, Pink1, Litaf, BC028528, Pbxip1, Pmp22, Sash1, Rgs2, Aplp2, Cttnbp2nl, Csnk1e, Pla2g15, Atp6v1b2, Mertk, Ccl6, Slfn5, Itgam, Epb41l2, Lat2, Tecpr1, Cxcl16, Psen2, AC149090.1, Zfand5, Cxxc5, Tns3, Tmem176b, Ptpn1, Il10ra, Adam8, Calr, Rasa4, Anxa5, S100a1, Scn1b, Tmem140, Ophn1, Lgals3, Abi3, Slco2b1, Rnf149, Tifa, Lpcat2, Zfp36, Fabp3, Pgd, Lsp1, Cst7 |
