# Supplementary figures and images for: Culture shock: microglial heterogeneity, activation, and disrupted single-cell microglial networks in vitro
Source: Mol Neurodegener. 2022 Mar 28;17:26. doi: 10.1186/s13024-022-00531-1 (PMC8962153; doi:10.1186/s13024-022-00531-1)

Supplementary Figure 1

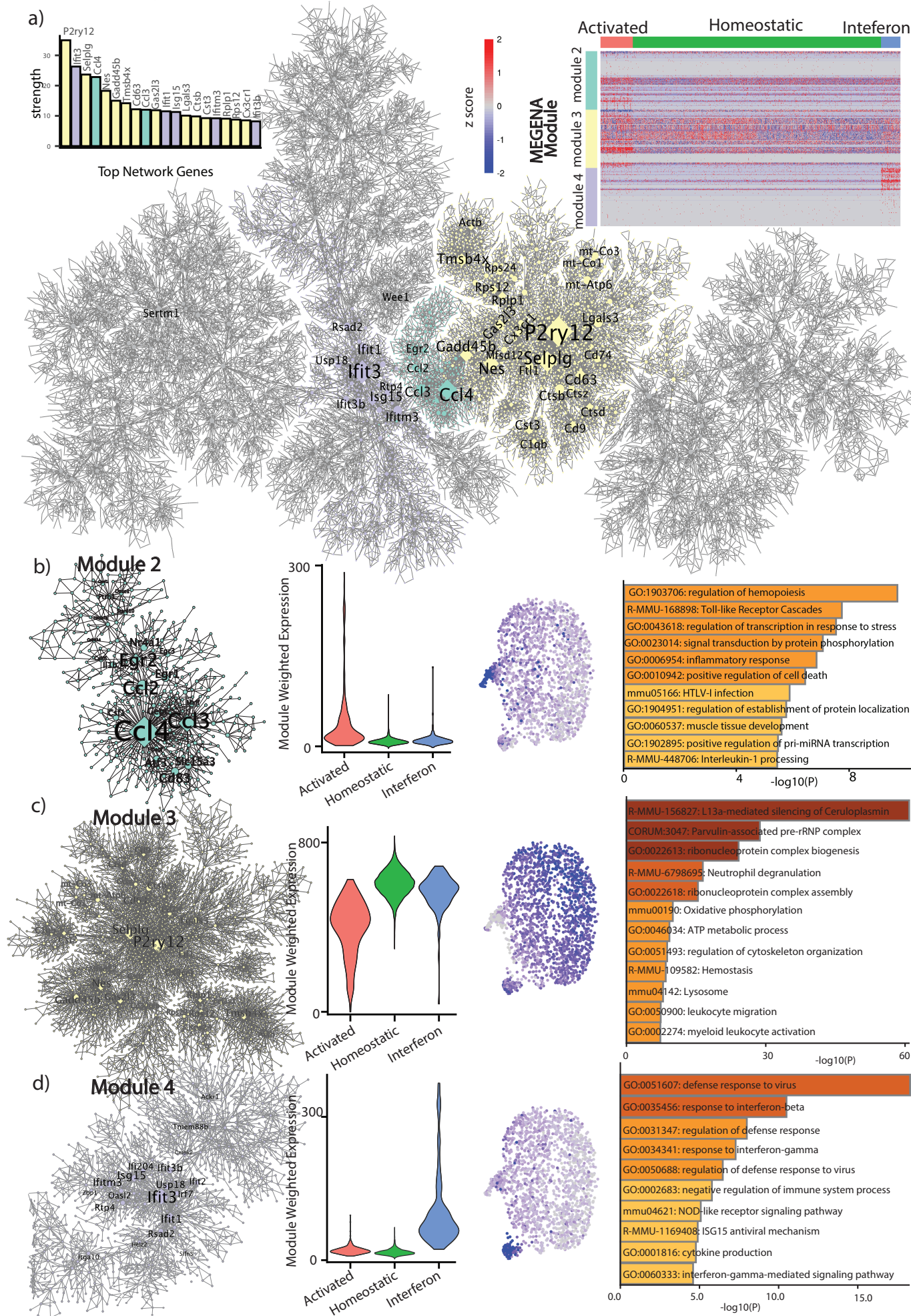

Supplement: Supplementary file 2 — Additional file 2: Figure S1. MEGENA network analysis of freshly isolated cells establishes a reference transcriptional network for microglia. (a) Transcriptional regulatory network for freshly isolated microglia inferred by MEGENA. Nodes represent genes, and an edge between nodes represents the significant correlation of those two genes. Bar graph shows relative strength of these top 20 nodes and highlights the module to which each gene belongs. Heatmap shows the scaled expression of the top 100 genes of each of the 3 core MEGENA modules. Heatmap is represented by a downsampled 2000 of the freshly isolated cells. (b) Subnetwork of module 2. Weighted module expression is calculated by calculating the sum of the expression of each gene in the module, weighted by its strength in the network, and signed by whether that gene has positive or negative correlation with the largest hub of that module. Plotting the module weighted sum in a violin and feature plot shows strong, significant association of module 2 with the activated cluster (two-tailed t-test between activated vs homeostatic cluster, p-value = 1.93e-15). Metascape gene ontology (GO) enrichment suggests that this module is related to inflammatory response and the transcriptional response to stress. (c) Subnetwork of module 3, containing many homeostatic microglia markers. Weighted module expression is highest in the homeostatic cluster and significantly decreases in cells from the activated cluster (two-tailed t-test between activated vs homeostatic cluster, p-value = 4.34e-60). Metascape shows that pathways enriched for this module include ribonucleoprotein complex formation, functioning of the lysosome, and immune pathways like neutrophil degranulation and myeloid activation. (d) Network of the subnetwork for module 4, which contains many interferon-related genes. Module weighted sum is significantly increased in the interferon cluster compared to the homeostatic cluster (two-tailed t-test, p-value = 7.65e-23 [file 13024_2022_531_MOESM2_ESM.pdf]

Supplementary Figure 2

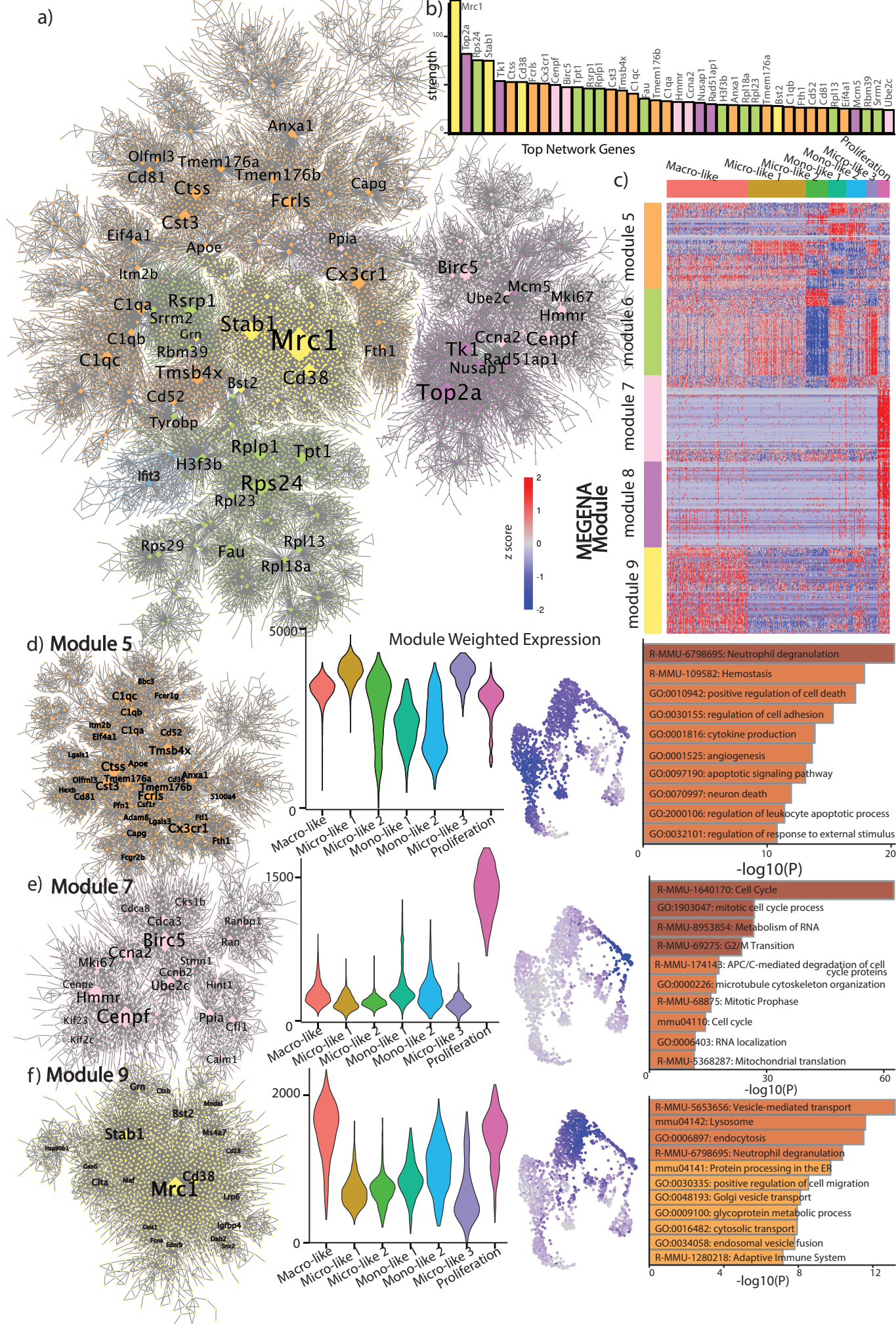

Supplement: Supplementary file 3 — Additional file 3: Figure S2. In vitro microglia networks are disrupted and dissimilar to the freshly isolated network. (a) Transcriptional regulatory network for astrocyte-plated in vitro cells inferred by MEGENA. Top 50 nodes are labelled, and nodes are colored corresponding to the 5 core modules identified by MEGENA clustering analysis. (b) Bar graph of the top 40 nodes shows from a relative strength between nodes. (c) Heatmap of the scaled expression of the top 75 genes of each core module across 2000 downsampled cells. Modules strongly associate with specific clusters. Module 9 contains genes highly expressed in the macrophage-like cluster. Module 5 contains genes associated with the microglia-like 1, and monocyte-like 1 and 2 clusters. Module 6 contains hubs of ribosomal genes and is downregulated in microglia-like cluster 2 and upregulated in microglia-like cluster 3. Modules 7 and 8 contain cell cycle genes associated with proliferation cluster. (d) Subnetwork of module 5, a heterogenous module that contains a variety of hubs. Hubs like Cx3cr1, Fcrls, and Fcer1g are markers of resting microglia, while hubs like Apoe, Lgals1, Lgals3 are activation markers. Smaller hubs like Anxa1 and S100a4 are unique to monocyte-like cells. The weighted module expression of this module is strongest in the microglia-like 1 cluster, but expression of this module is robust across all clusters. Metascape GO enrichment shows enrichment for immune and cell death processes. (e) Subnetwork of module 7, a cell cycle module whose module weighted expression is significantly higher in proliferation cluster 6 (two-tailed t-test, p-value = 1.62e-166). Metascape analysis of this clusters shows enrichment of cell cycle processes. (f) Subnetwork of module 9, with hubs Mrc1 and Stab1 that are unique markers of macrophages. Weighted module expression of this module is expressed robustly in all clusters, but is highest in macrophage-like cluster 0 (two-tailed t-test, p-value = 8.23e-7). Metasca [file 13024_2022_531_MOESM3_ESM.pdf]

Figure S3

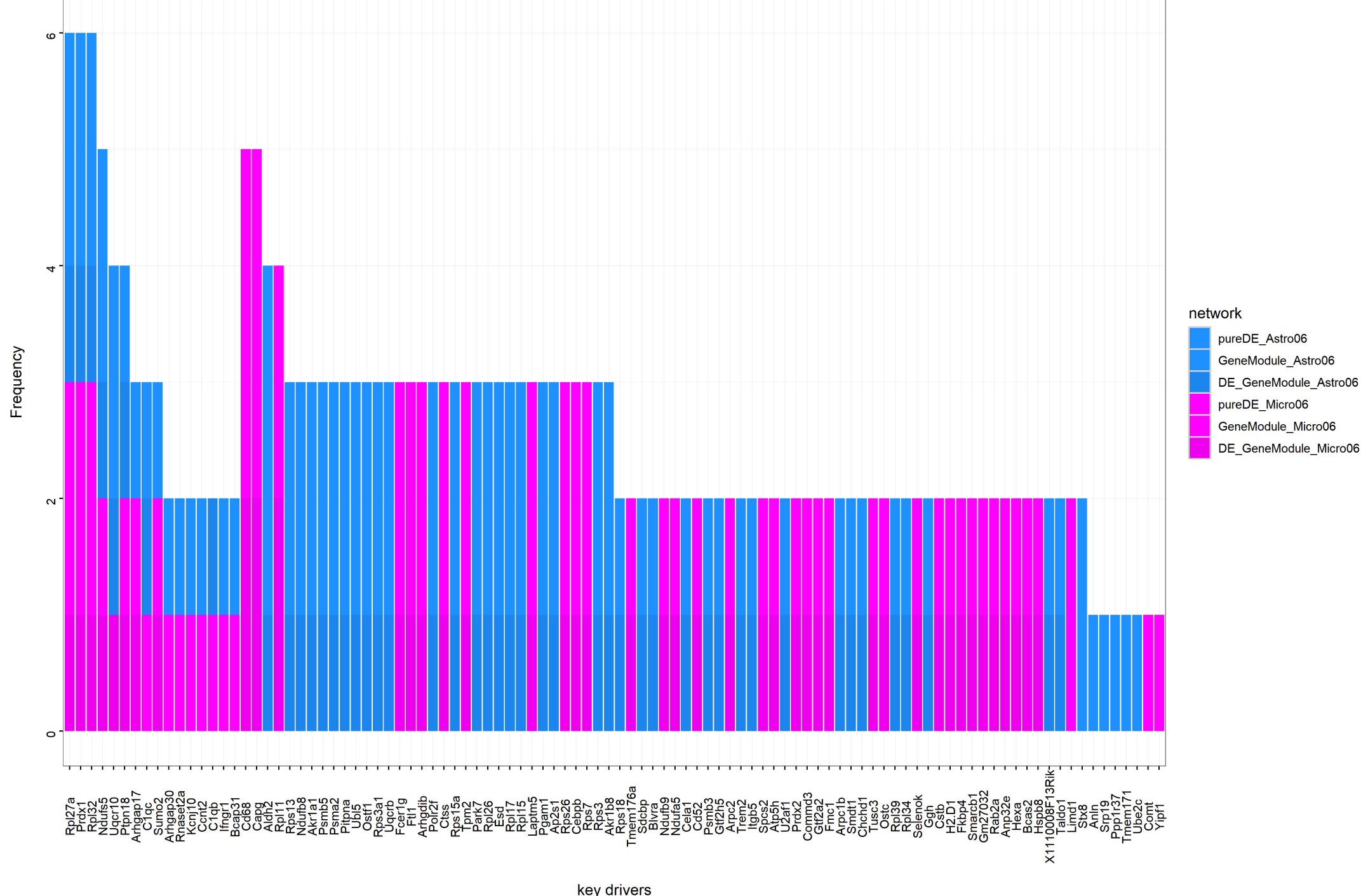

Supplement: Supplementary file 4 — Additional file 4: Figure S3. Key drivers of the cultured microglia phenotype. Top 100 of 444 total key drivers with prioritized rank derived from the astro-plated microglia and micro-isolated microglia networks. [file 13024_2022_531_MOESM4_ESM.pdf]
